# Predicting the effects of climate change on the cross-scale epidemiological dynamics of a fungal plant pathogen

**DOI:** 10.1038/s41598-022-18851-z

**Published:** 2022-09-01

**Authors:** Ian F. Miller, Juliana Jiranek, Mckenna Brownell, Sarah Coffey, Barrett Gray, Maria Stahl, C. Jessica E. Metcalf

**Affiliations:** 1 Department of Ecology and Evolutionary Biology, Princeton University, Princeton, USA; 2 Rocky Mountain Biological Laboratory, Crested Butte, USA; 3 Department of Biology, University of Virginia, Charlottesville, USA; 4 Department of Wildland Resources, Utah State University, Logan, USA; 5 School of Public and International Affairs, Princeton University, Princeton, USA

**Keywords:** Ecological epidemiology, Climate-change ecology, Ecological modelling

## Abstract

The potential for climate change to exacerbate the burden of human infectious diseases is increasingly recognized, but its effects on infectious diseases of plants have received less attention. Understanding the impacts of climate on the epidemiological dynamics of plant pathogens is imperative, as these organisms play central roles in natural ecosystems and also pose a serious threat to agricultural production and food security. We use the fungal ‘flax rust’ pathogen (*Melampsora lini*) and its subalpine wildflower host Lewis flax (*Linum lewisii*) to investigate how climate change might affect the dynamics of fungal plant pathogen epidemics using a combination of empirical and modeling approaches. Our results suggest that climate change will initially slow transmission at both the within- and between-host scales. However, moderate resurgences in disease spread are predicted as warming progresses, especially if the rate of greenhouse gas emissions continues to increase at its current pace. These findings represent an important step towards building a holistic understanding of climate effects on plant infectious disease that encompasses demographic, epidemiological, and evolutionary processes. A core result is that neglecting processes at any one scale of plant pathogen transmission may bias projections of climate effects, as climate drivers have variable and cascading impacts on processes underlying transmission that occur at different scales.

## Introduction

Understanding the epidemiology of plant pathogens in a changing world is important, as these organisms significantly hinder agricultural production^1,2^, threaten food security^3,4^, and play a crucial role in mediating ecosystem processes^5,6^. Environmental conditions have long been known to be important determinants of disease spread in plant pathosystems^7^, and climate change is increasingly recognized as a dominant driver of current and future trends^8^. However, there is a surprising knowledge gap surrounding the effects of climate on plant pathogen epidemiology, i.e., the dynamics of disease spread within populations.

Most previous studies of plant pathogen spread in the context of climate change have focused on disease severity, infection risk, or the geographic distribution of disease^9^. However, anticipating and mitigating the changing burden of pathogens on natural or agricultural plant populations requires an epidemiological perspective^10,11^. Logically, the impact of disease on the mortality, fecundity, biomass, or agricultural productivity of a population depends not only on the consequences of infection for individual plants, but also on the proportion of plants that become infected. Furthermore, the details of within- and between-host processes will shape future impacts infectious disease, particularly if climate change has cascading effects across scales of transmission. This could occur if, for example, warmer temperatures speed disease progression within infected plants resulting in increased transmission, and also increase the odds that a healthy plant becomes infected when challenged.

Of the few studies that have investigated effects of climate change on plant disease spread at the population scale, all (to our knowledge) have taken a statistical approach, directly extrapolating relationships between climate variables (e.g., temperature) and epidemiological variables (e.g., prevalence) to make projections. None have formally encompassed the underlying within-host processes inherent to pathogen transmission, evaluated how these are affected by climate, and integrated across these within-host processes to generate a mechanistic (or semi-mechanistic) projection of the effects of climate on epidemiological variables like pathogen prevalence. While previous studies are tremendously useful for detecting broad scale effects of climate on disease dynamics^12,13^, generating robust predictions requires epidemiological models that both capture climate effects on different scales of transmission and link these processes in one predictive framework. Such models are widely applied in the fields of human and animal infectious disease dynamics^14^, but are underutilized in the realm of plant infectious disease, with a focus typically limited to disease severity^15^.

In this study, we use *Linum lewisii*, a sub-alpine wildflower, and *Melampsora lini*, its fungal pathogen, as a model to investigate the effects of climate change on cross-scale transmission processes using a semi-mechanistic epidemiological model. This ‘flax rust’ system^16^ is a representative example for the effects of climate change on the epidemiology of fungal plant pathogens, and also parallels many aspects of flax rust disease in agricultural systems. Our findings indicate that disease severity and prevalence within individual epidemics could decrease in the short term, but eventually rebound due to continued climate change.

## Results

### Study system

**Figure 1:**
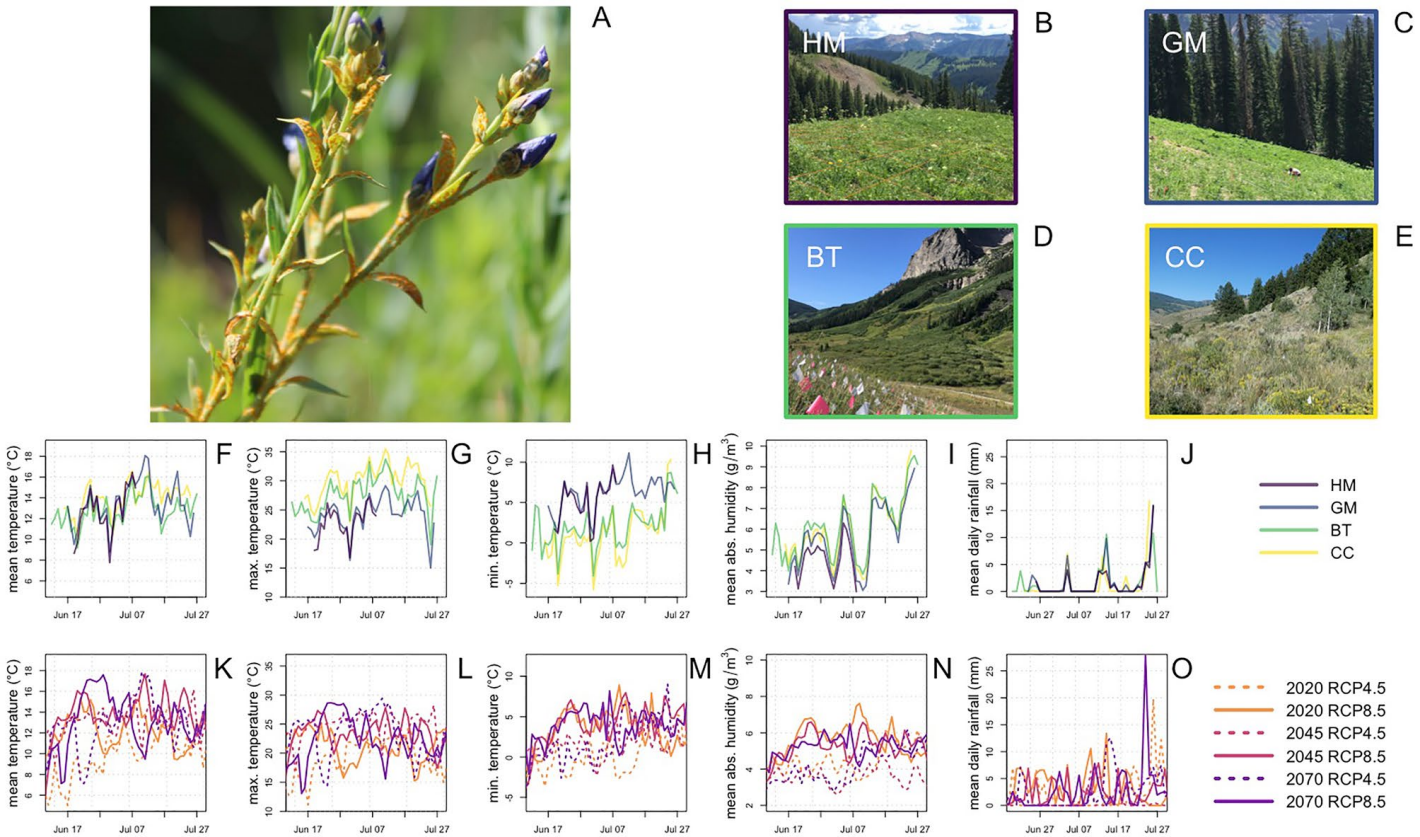
The flax rust study system. Panel (**A**) shows a *L. lewisii* plant infected with *M. lini.* Panels (**B**–**E**) shows photographs of the ‘high meadow’, ‘gothic mountain’, ‘bus turnaround’ and ‘cement creek’ (hereafter HM, GM, BT, CC) sites. These sites are located at approximately 2440 m, 2940 m, 3200 m, and 3410 m elevation respectively. Panels (**F**–**J**) show observed weather data collected at each site over the duration of the study period, which occurred in June and July of 2020. Panels (**K**–**O**) show weather data projected for the area surrounding the BT study site. These projections were generated for future climate scenarios encompassing the RCP 4.5 (dashed lines) and 8.5 (solid lines) emissions pathways for the years 2020 (year of observations, orange lines), 2030, 2040, 2045 (magenta lines), 2050, 2060, and 2070 (purple lines). The RCP 4.5 projections correspond to an intermediate scenario in which emissions begin to decline by approximately 2050, and the RCP 8.5 projections correspond to a more pessimistic scenario in which emissions increase through 2100^17^.

We aim to predict how climate change might alter the within and between host transmission dynamics of fungal plant pathogens using the flax rust model system. The host, *L. lewisii* (Lewis flax)*,* is a short-lived perennial plant distributed across the western United States, and the pathogen, *M. lini*, is rust fungus. *M. lini* urediniospores infect the stems and leaves of *L. lewisii*, forming bright yellow-orange pustules (Fig. 1A). The spores propagated by these pustules are dispersed by the wind both within and between plants. This infection cycle repeats continuously over the course of a growing season^16^ until plant tissue senescence truncates transmission. The flax rust pathosystem is an excellent model for studying the effects of climate on the epidemiology of plant pathogens because *M. lini* completes all of its life stages on its flax host^16^, transmission occurs across a wide range of environmental conditions, and infection is easily identified visually in situ.

To observe how processes of disease spread play out in different climate conditions, we studied four populations of *L. lewisii* in the vicinity of Crested Butte, Colorado, USA. These sites span an elevation gradient of roughly 1000 m (Fig. 1B–E; Supplementary Fig. S1). At each site we monitored temperature, humidity, and rainfall (Fig. 1F–J). The large variation in these weather metrics between sites and across time allows for environmental drivers of transmission processes to be quantified via statistical modeling. We begin by analyzing the effects of climate change on the within-host processes of fungal replication, infection intensity progression, and plant growth. Next, we consider climate effects on transmission, linking spore deposition and environmental conditions to infection risk. Finally, we present a spatiotemporal epidemiological model, validate it against observed epidemiological patterns, and use it to project climate effects on population level patterns of disease spread.

### Within-host processes

#### Replication rate

**Figure 2:**
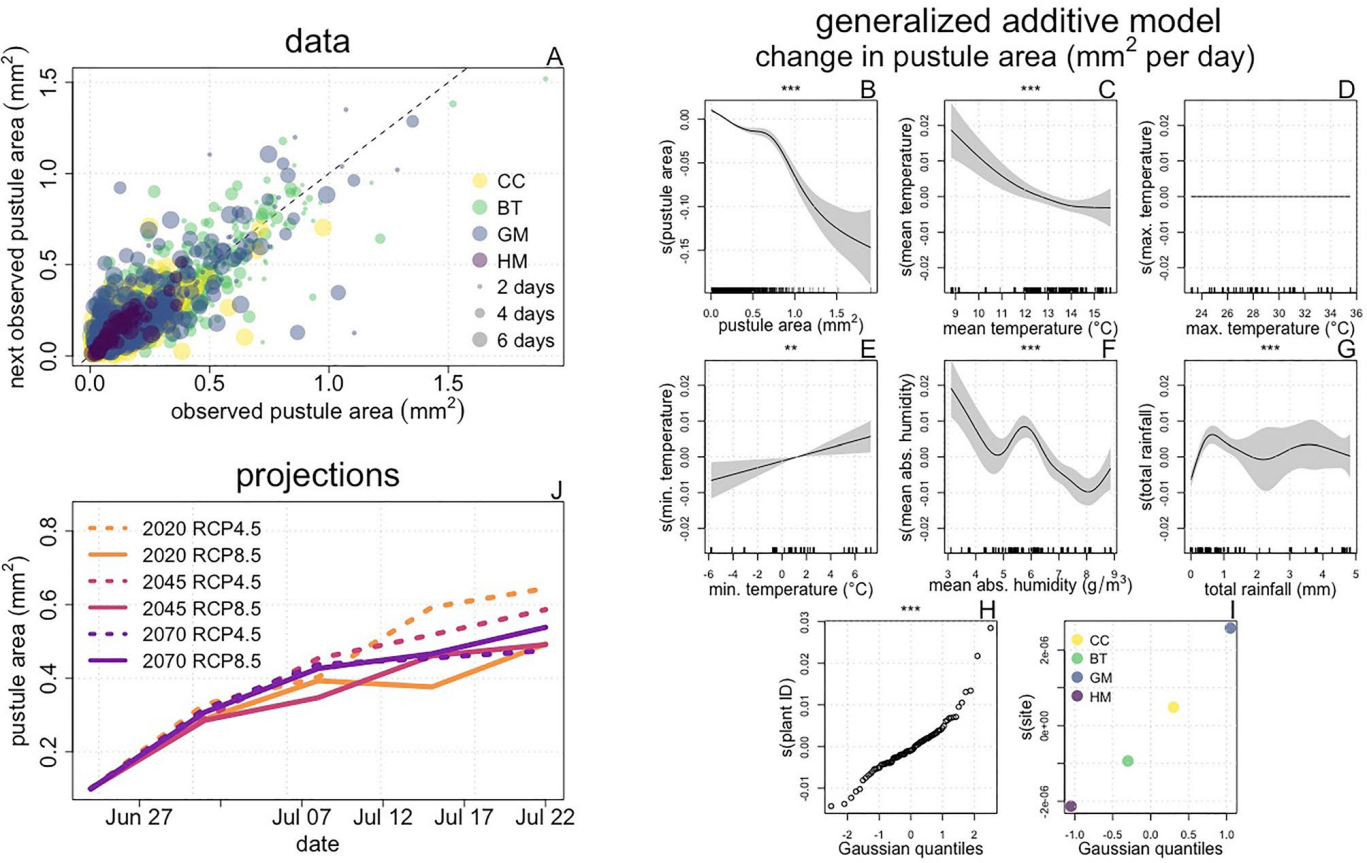
The effects of climate change on *M. lini* replication rate. Data: We made 3319 observations of pustule area from 1434 individual pustules located on 84 diseased focal plants. Panel (**A**) shows this data, with each point representing one observation. Color indicates study site, and size represents the elapsed time between observations. While changes in pustule area capture pustule growth, they also capture processes that decrease pustule size (e.g. wind and rain damage, which may potentially contribute to spore dispersal), so both positive and negative changes are expected. The dashed line shows y = x to illustrate where increases (points above the line) and decreases (points below the line) in pustule area occur. Generalized additive model: We fit a GAM with an identity link function to this data, using change in pustule area per day as the response variable and smooth terms for weather variables and pustule area as predictors. We also included source plant identity and study site as random effect smooths to account for the nested nature of our data. Panels (**B**–**I**) show the value of the fitted smooths (i.e. ‘s(x)’) for different values of the predictor variables. Symbols above plots show the significance of smooth terms, with *** indicating p < 0.001, ** indicating p < 0.01, and * indicating p < 0.05. In the model fitting and selection procedure, smooth terms lacking explanatory power are set to have no effect and thus are functionally removed from the model, as seen in panel (**D**). In panels (**B**–**E**), lines show the fitted value of the smooth, and shading represents ± 2 standard errors. Ticks along the horizontal axis represent observed weather metrics. In Panels (**H**) and (**I**), points show the fitted values of random effects vs. the quantile of their fitted value. Projections: Using the fitted GAM model, we 100 simulated trajectories of pustule growth for a small pustule (starting with area 0.1 mm^2^) at the four study sites for each of six future weather data sets. Panel (**J**) shows the means of trajectories simulated for the GM site. These qualitatively match results for other sites (Supplementary Fig. S4).

Replication rate is a key process underlying the spread of *M. lini*, as it produces the urediniospores necessary for disease proliferation. Understanding the effects of weather conditions on this trait is important for determining how and why changes in infection intensity progression might occur in future climate scenarios. We tracked longitudinal changes in pustule area (Fig. 2A; Supplementary Fig. S2) as a proxy for fungal replication rate. To infer the effects of environmental conditions on pustule growth, we fit a generalized additive model^18^ (GAM) of change in pustule area per day to this data, including smooth predictor terms for five climate variables: mean, maximum and minimum temperature; mean absolute humidity; and mean daily rainfall. These variables were selected because they have been previously found to affect either pustule growth or disease progression^13,19^. We also included random effects for study site and the identities of the ‘source plants’ on which the pustules were observed.

The fitted GAM (Fig. 2B–I) points to the presence of an allometric effect of pustule size, with an increase in size expected for small pustules, and a decrease in size expected when pustule area exceeds approximately 0.2 mm^2^ (Fig. 2B). Cooler mean temperatures and warmer minimum temperatures produced the largest positive effect on change in pustule area (Fig. 2C–E). Absolute humidity values less than ~ 6.5 g/m^3^ promoted pustule growth, while more humid conditions impeded pustule growth (Fig. 2F). A small amount of rainfall (up to ~ 0.5 mm per day) promoted pustule growth (Fig. 2G), but the model indicated uncertainty about the effects of increased rainfall for greater amounts. In most cases, the identity of the source plant had a minimal effect on pustule growth. These fitted effects had a linear relationship with theoretical gaussian quantiles, indicating that they are approximately normally distributed (Fig. 2H). To infer how climate change might affect patterns of *M. lini* replication, we simulated pustule area trajectories from the fitted GAM using weather data projected for future climate scenarios (see Fig. 1K–O). We used projected climate data rather than observed weather data to conduct simulations for 2020 to avoid biases introduced by data source in comparisons of results across years. The simulated growth trajectories indicate that in the RCP 4.5 emissions scenario, *M. lini* replication rate will gradually slow over time as climate change progresses. Replication rates were projected to remain relatively constant over time in the RCP 8.5 scenario, with a very slight positive trend from 2040 to 2070 (Fig. 2J, Supplementary Fig. S3).

#### Infection intensity progression

**Figure 3:**
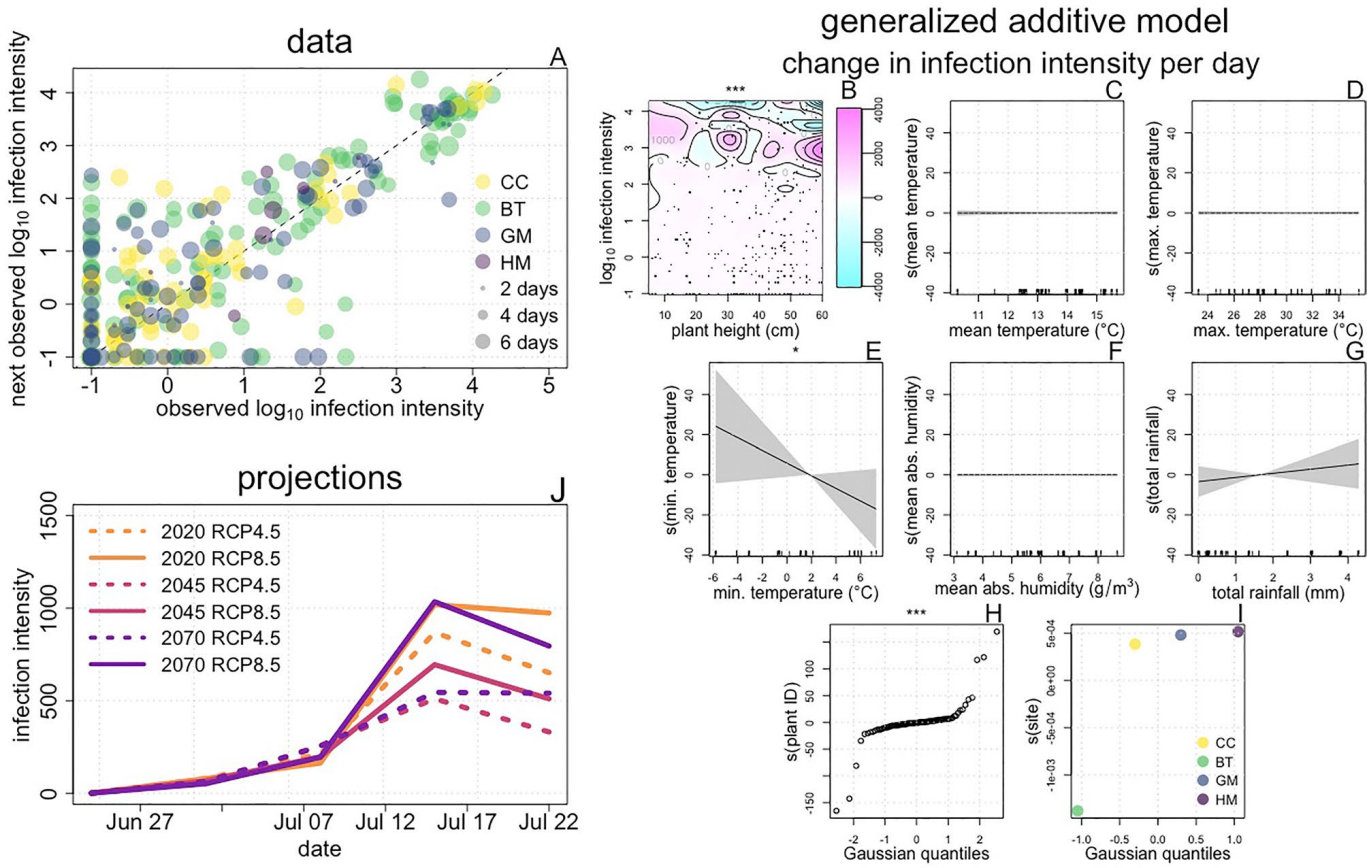
The effects of climate change on the progression of *M. lini* infection intensity. Data: Across 90 diseased focal plants, we made 313 observations of infection intensity (Panel **A**). Both increases and decreases in infection intensity are expected, as both the proliferation of infection and the deterioration of infected tissue may occur. Panel (**A**) shows log_10_ transformed observed data, with each point representing one observation. Point color indicates study site, and point size represents the elapsed time between observations. The dashed line indicates y = x to illustrate where increases and decreases in infection intensity occur. Generalized additive model: We fit a GAM of change in infection intensity per day, using an identity link function. Predictors included smooth terms for environmental conditions, a full tensor smooth product (which captures both individual and joint effects of predictors) of log_10_ infection intensity and plant height, and random effect smooths for plant identity and site. We expect height and infection intensity to have a joint effect for two reasons. First, height can partially constrain infection intensity by limiting the amount of tissue that can be infected. Second, quantitative resistance, which could slow the spread of infection within a plant, could be correlated with plant size, as leaf age influences pustule development^20^. Panel (**B**) shows the fitted tensor product, with colors and contours indicating the tensor value (analogous to smooth value) for different combinations of plant height and log_10_ infection intensity. Points indicate observed data. Panels (**C**–**I**) show the fitted smooth terms, with axes, lines, and shading as in Fig. 2B–G. Panels (**H**) and (**I**) show fitted random effects, with points and axes as in Fig. 2H–I. Symbols above plots show the significance of smooth terms, with *** indicating p < 0.001, ** indicating p < 0.01, and * indicating p < 0.05. Projections: We simulated 100 trajectories of infection intensity for a lightly infected plant (infection intensity 1) of approximately average height (25 cm) at each site for six future weather data sets using the fitted GAM. The means of these trajectories, simulated for the GM site, are shown in panel (**J**). These results qualitatively match those simulated for other sites (Supplementary Fig. S7).

Next, we turn to the effects of climate on the progression of infection intensity. We tracked infection intensity in diseased focal plants (Fig. 3A; Supplementary Fig. S5), calculating a unitless infection intensity score (designed to synthesize available measures to quantitatively capture the overall severity of infection) as the product of (1) the number of infected stems, (2) the average length of infected tissue per stem, and (3) the average number of pustules per leaf. Using this data, we again fit a GAM to infer how environmental conditions affect the rate of infection intensity progression. The fitted GAM (Fig. 3B–I) of change in infection intensity per day indicates that minimum temperatures less than 2 °C have a positive effect on infection intensity progression (i.e. they increase infection intensity), while higher minimum temperatures have a negative effect (i.e. they decreased infection intensity, Fig. 3E). Increasing rainfall had a small positive effect (Fig. 3I). The tensor product (Fig. 3B) revealed a complex interaction between infection intensity and height. All combinations of plant height and log_10_ infection intensity had a positive effect on infection intensity progression when log_10_ infection intensity was low (i.e. less than ~ 2.5), perhaps due to the high availability of plant tissue for the fungus to colonize. For heavily infected plants (log_10_ infection intensity between ~ 2.5 and ~ 4), different combinations of plant height and infection had either strong positive or weak negative effects, reflecting the diversity of observed infection intensity trajectories (Supplementary Fig. S5). For very heavily infected plants (log_10_ infection intensity greater than ~ 4), most combinations of infection intensity and plant height had strong negative effects. This is potentially due to the rate of pustule degradation exceeding the rate of new tissue colonization when most tissue has been infected. While in most cases plant identity had a weak effect on infection intensity progression, the non-linear relationship between fitted effects and theoretical gaussian quantiles reflects a heavy-tailed distribution, indicating that infection may progress much faster or slower in some plants than in others (Fig. 3H).

We generated projections about how climate change might affect the progression of infection intensity by simulating infection intensity trajectories using the fitted GAM and future weather data projected for different climate scenarios. We found that climate change will initially slow the rate of infection intensity progression. Following this, a rebound in infection intensity progression will occur, although infection intensity levels are not projected to surpass the 2020 benchmark. Peak infection intensity was lower for RCP 8.5 scenarios than for RCP 4.5 scenarios (Fig. 3J; Supplementary Fig. S6).

#### Plant growth

**Figure 4:**
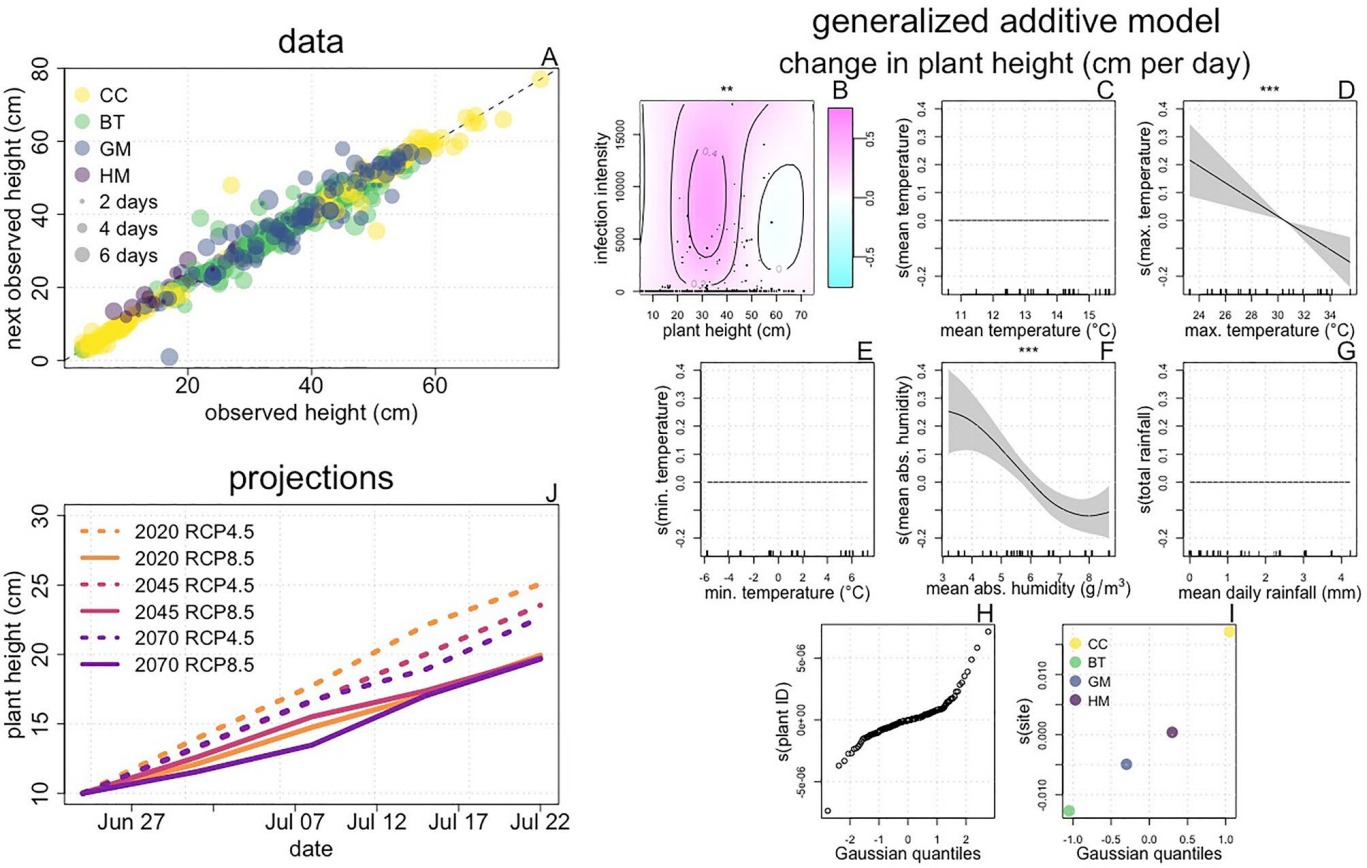
The joint effects of climate change and infection on *L. lewisii* growth. Data: We made a total of 596 observations of plant height from 175 focal plants (panel **A**). Points show individual observations with color indicating site and size indicating elapsed time. Unsurprisingly, we observed both increases and decreases in plant height as plant condition changed over the course of the observation period. The dashed line illustrates y = x to show where increases and decreases occurred. Generalized additive model: We fit a GAM to this data, using change in plant height per day as the response variable, and smooth terms for weather variables as predictors along with a full tensor product of height and infection intensity. Plant identity and site were included as random effect smooths. Panel (**B**) shows the fitted tensor, with contours, shading, and points as in Fig. 3B. Panels (**C**–**G**) show fitted smooth terms with axes, lines, and shading as in Fig. 2B–G. Panels (**H**) and (**I**) show fitted random effects with points and axes as in Fig. 2H–I. Symbols above plots show the significance of smooth terms, with *** indicating p < 0.001, ** indicating p < 0.01, and * indicating p < 0.05. Projections: We simulated 100 growth trajectories for a small plant (starting with a height of 10 cm) at each site for six future weather data sets using the fitted GAM. The lines in panel (**J**) show means of the trajectories simulated for the GM site. Qualitatively similar patterns were observed in simulations performed for other sites (Supplementary Fig. S10).

Impacts of climate variables on fungal growth alone do not capture the range of impacts of fungal populations. The effects of climate on plant growth are also relevant due to the role plant height plays in (i) regulating the progression of infection intensity (Fig. 3B), (ii) determining patterns of spore dispersal, and (iii) modulating the risk of infection (see “Transmission” section below for background on ii and iii). Using plant height data collected from infected and uninfected focal plants (Fig. 4A; Supplementary Fig. S8), we fit a GAM to characterize how environmental conditions and infection intensity affect plant growth. The fitted model (Fig. 4B–I) indicates that the rate of plant growth declines as maximum daily temperature and absolute humidity increase, and that there is no significant stunting effect of infection.

To infer how climate change might affect patterns of plant growth in *L. lewisii*, we simulated growth trajectories using the fitted GAM and future weather data projected for future climate scenarios. These simulations do not account for the effect of increasing CO_2_ levels on plant growth^21^, and reflect only the impacts of environmental conditions. Broadly, growth rates are projected to be higher in the RCP 4.5 scenario compared to the RCP 8.5 scenario. Climate change will slow the rate of plant growth over time in the RCP 4.5 scenario, but in the RCP 8.5 scenario, growth rates are expected to remain approximately constant except for a transient increase in 2030. (Fig. 4J; Supplementary Fig. S9).

### Transmission

After inferring the effects of environmental conditions and climate change on within-host processes, we set about connecting the within-host spread of disease to between-host patterns of transmission. First, we collected data on the spread of *M. lini* within *L. lewisii* populations, tracking the spread of infection through space and time at each of the four study sites. We began by mapping the location of each plant in a 10 by 20 m transect at each study site. We then inspected each plant for the presence of infection approximately every seven days.

Next, we sought to connect infection intensity and plant height to spore dispersal. We deployed spore traps in arrays around diseased plants, and counted the number of *M. lini* spores caught in each trap. Using this data, observed patterns of wind speed and direction, and measurements of source plant infection intensity and height, we parameterized a tilted gaussian plume model^22^ (TGPM) describing spatial patterns of spore dispersal (see “Methods”, Supplementary Figs. S11–13). As the TGPM predicts the number of spores per mm^2^ deposited at the coordinates of a target plant, rather than the number of spores coming into contact with the plant, we present spore dispersal as a unitless metric that captures the relative intensity of spore deposition experienced by a plant.

**Figure 5:**
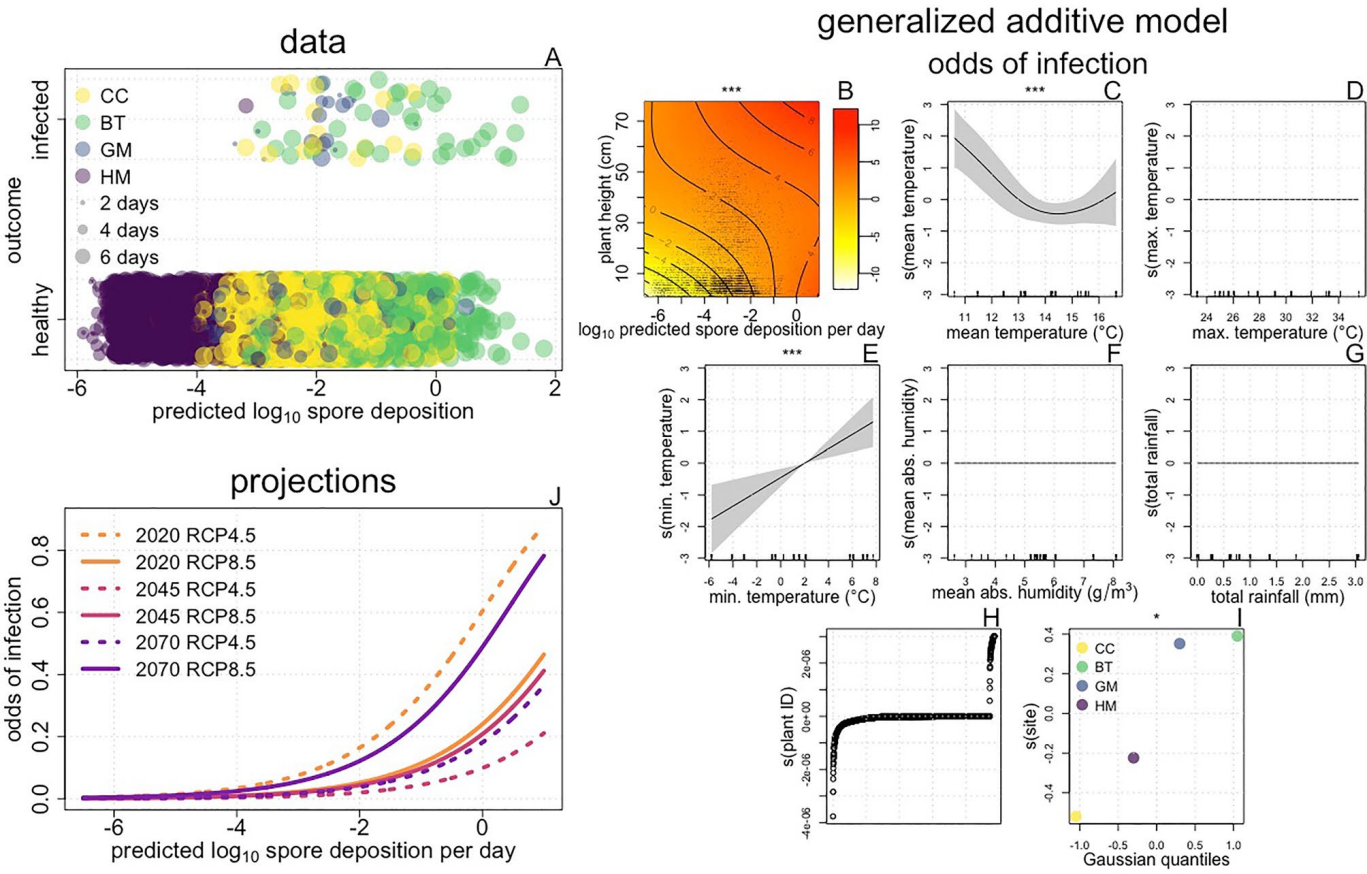
The effects of climate and spore deposition on infection risk. Data: We predicted the total spore deposition experience by each uninfected plant at each study site over the periods between observations of infection status. We made these 9142 predictions using the TGPM in conjunction with observed wind speed, the locations of infected plants, and the infection intensities and heights of those plants. Points in panel (**A**) show infection outcome vs log_10_ predicted spore deposition, with the color and size of the points representing study site and the time between observations. Generalized additive model: We fit a GAM to this data using infection outcome as the response variable and assuming a binomial response distribution. We used a complementary log–log link function and included an offset of log time to account for variation in the length of the observation period. Predictors included smooth terms for weather variables, random effect smooths for plant identity and site, and a full tensor product of log_10_ predicted spore deposition per day and plant height. Panel (**B**) shows the fitted tensor, with contours, shading, and points as in Fig. 3B. Panels (**C**–**G**) show fitted smooth terms with axes, lines, and shading as in Fig. 2B–G. Panel (**H**) shows fitted random effects for plant identity plotted in the order of their magnitude. Panel (**I**) shows fitted random effects for site with points and axes as in Fig. 2I. Symbols above plots show the significance of smooth terms, with *** indicating p < 0.001, ** indicating p < 0.01, and * indicating p < 0.05. Projections: Lines in panel (**J**) show the relationship between log_10_ predicted spore deposition per day and the odds of infection (for a plant with a height of 25 cm at the GM site) projected from the fitted GAM for the time period spanning July 7th–July14th. We replicated these predictions across weather data sets projected for six climate scenarios.

Finally, we inferred the relationships between environmental conditions, spore deposition, and infection risk. We used the TGPM to predict the spore deposition experienced by each healthy plant in each study population between observations of the infection status (Fig. 5A). We then modeled the relationship between log_10_ predicted spore deposition, environmental conditions, and infection occurrence using a GAM (Fig. 5B–I). The fitted GAM indicated the presence of a thermal optimum for transmission, with the odds of transmission increasing with warmer minimum temperatures (Fig. 5C), but decreasing with warmer mean temperatures (Fig. 5E). Unsurprisingly, the odds of transmission rose with increasing spore deposition (Fig. 5J). For a given level of log_10_ predicted spore deposition, infection odds generally increased with plant height (Fig. 5B; Supplementary Fig. S14). This is intuitive, because the TGPM predicts spore deposition for a point in space, but larger plants represent bigger ‘targets’ for spore deposition, and should experience greater actual spore deposition for a given level of predicted spore deposition. Supplementary Figure S14 illustrates the details of the relationships between plant height, spore deposition, and time inferred by the fitted GAM.

We investigated how climate change might affect infection risk by simulating the fitted GAM using weather data projected for future climate scenarios. We observed that climate change is expected to initially lower infection risk, but that a resurgence in risk is expected as climate change continues. The resurgence in risk projected for 2070 exceeds the 2020 baseline in the RCP 8.5 scenario but not in the RCP 4.5 scenario. These results indicate that in the short term, climate change might lower infection risk, but this reduction will be transient if emissions continue to increase over time (Fig. 5J, Supplementary Fig. S15).

### Epidemiological model

**Figure 6:**
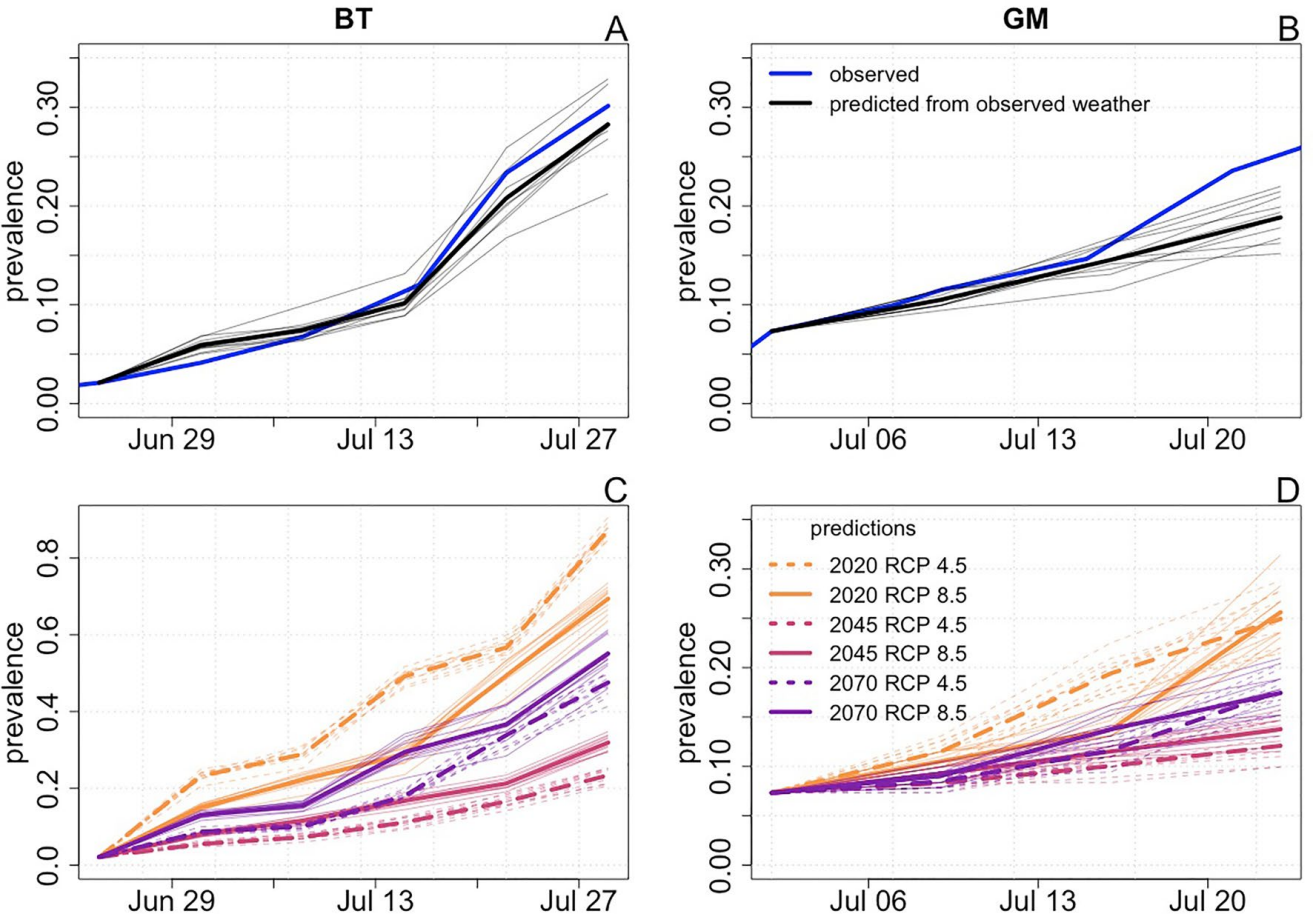
The effects of climate change on the epidemiology of flax rust disease. Panels (**A**) and (**B**) show the observed patterns of infection prevalence at the BT and GM sites (blue lines) along with prevalence simulated from 10 runs of the spatiotemporal epidemiological model using observed weather data (thin black lines). The thick black lines show the mean of the 10 simulations. Panels (**C**) and (**D**) show patterns of infection prevalence at the BT and GM sites simulated for six future climate scenarios. We simulated 10 epidemics at each site for each climate scenario. Thin lines show prevalence predicted from individual simulations, and thick lines show mean prevalence across 10 simulations for a given climate scenario.

In the final step of our analysis, we extended our inferences about the effects of environmental conditions on within-host processes and transmission to generate predictions about the effects of climate change on the spread of infection at the population scale. To accomplish this, we constructed a spatiotemporal epidemiological model using the parameterized TGPM and fitted GAMs of infection odds and within-host processes. We validated the model by simulating epidemics at the BT and GM sites using observed weather data and confirming that predictions qualitatively recapitulated observed epidemiological patterns (Fig. 6A, B). Epidemics simulated from observed weather data at the GM site slightly underpredict prevalence by the end of the observation period (Fig. 6B). A potential explanation for this slight discrepancy could be that infected plants observed just outside of the transect may have contributed to spore deposition in the transect. To infer the effects of climate on population level disease spread, we again simulated epidemics at the BT and GM sites, this time using weather data sets projected for future climate scenarios. These results represent projections of how the observed epidemic might have progressed if it had played out in future climate conditions.

These results of these simulations (Fig. 6C, D; Supplementary Fig. S16) reveal that climate change is likely to slow the spread of disease in the short term, but that continued climate change will likely lead to a resurgence in disease spread, albeit one that falls short of current levels. This pattern was observed for both the RCP 4.5 and 8.5 emissions scenarios. Our simulations predict greater reductions in the pace of disease spread in the short term for the RCP 8.5 scenario, as prevalence in 2020–2040 was projected to be higher in this emissions pathway as compared to the RCP 4.5 pathway. However, increased emissions are projected to amplify the resurgence in disease spread, as projections for the RCP 8.5 scenario match or exceed those for the RCP 4.5 scenario by 2070.

## Discussion

Climate change is predicted to drive changes in the dynamics of epidemics across multiple scales of transmission in the flax rust plant pathosystem. At the within-host scale, warmer temperatures will hinder plant growth (although increasing atmospheric CO_2_ may have an opposite effect^21^), and infection will spread less rapidly, perhaps due to a decrease in the pathogen replication rate associated with rising mean temperatures. This will lead to a drop in infection risk due to both a reduction in spore dispersal and a decrease in the odds that a plant becomes infected when challenged with fungal spores. This combination of effects will decelerate the speed of epidemics at the population level. However, this slowing of transmission processes across scales is predicted to reverse as climate change continues, especially if greenhouse gas emissions increase (i.e. RCP 8.5 scenario). Rebounds in plant growth, infection intensity progression, infection risk, and epidemic pace are all predicted to occur after the initial deceleration.

A likely explanation for these predicted rebounds pertains to the details of the projected future weather data used to generate these predictions. June to early-July temperatures are warmer in the projected 2070 weather data compared to the projected 2045 data, but the opposite is true for mid-to-late July temperatures (Fig. 1K–M). Given the negative effect of increased temperature on disease spread at multiple scales, this difference in weather patterns is a probable explanation for disease resurgence. Broadly, this points to the importance of considering both the magnitude and timing of differences between current and future climate.

By using flax rust as a model for fungal plant pathogens in general, we can identify several important impacts of climate change on the dynamics of these diseases. While flax rust may not be a universally representative fungal pathogen, especially for systems with different thermal ecologies, our results can serve a much needed first approximation in most cases. In agricultural systems, there may be unexpected upsides associated with warming in the short term, as infection intensity and prevalence may decrease. In natural populations, the favorability of outcomes associated with climate change will be system-specific. Some species of conservation importance may benefit, such as Australian myrtle species (genus *Myrtaceae*) threatened by the rust fungus *Austropuccinia psidii*^23^, as increases in temperature slow disease development and epidemic spread^24^. In other systems, the threats posed by invasive species held in check by disease^25,26^ may increase. Ecosystem-level effects are likely to be complex, especially if multiple interacting pathogen and host species are affected.

We focused our investigation on the effects of climate change on patterns of transmission within a single epidemic. However, several factors not accounted for in our epidemiological model may also have significant direct and indirect effects on the relationship between climate and disease spread. These factors, including phenology, demography, and evolution, could impact the dynamics of both individual epidemics and multi-annual epidemic patterns. Incorporating these factors into long-term data epidemiological studies (spanning multiple epidemics) could allow for our predictions to be validated, improved, and extended beyond the timescale of a single epidemic. The effects of climate change on disease phenology (the duration and timing of the period during which environmental conditions render host condition and pathogen physiology adequate for disease spread^27^) could boost end-of-epidemic prevalence by lengthening the duration of conditions suitable for flax rust spread. Likewise, demographic feedbacks are important to consider^28^. Indeed, disease and climate effects on host mortality and demography have been linked to the presence of multi-annual epidemic cycles in the *Linum marginale*-*M. lini* flax rust system^13^. Analogous patterns exist in human systems such as measles, in which birth and vaccination rates, which in combination determine the rate of susceptible host recruitment, are linked to the periodicity of outbreaks^29^. Feedbacks between climate and evolutionary processes will also affect the dynamics of plant disease spread. Both ‘quantitative’ and ‘qualitative’ pathogen virulence and host resistance traits are important to consider in this context. ‘Qualitative’ traits jointly determine whether infection occurs, while ‘quantitative’ traits dictate the extent to which the pathogen exploits the host^30^. The *L. marginale*-*M. lini* flax rust system has been studied extensively as a model for the co-evolution of qualitative traits^31,32^, and associated epidemiological outcomes^33^. The effects of climate on replication rate may have consequences for selection on qualitative virulence, as these two traits are known to be linked^32^. Quantitative traits have also been studied in flax rust^20^, but have not been connected to epidemiological patterns. Our finding that warmer temperatures may slow infection intensity progression suggests that climate change might select for increased quantitative virulence in *M. lini* because a more aggressive host exploitation strategy could help the pathogen recoup transmission losses.

In this study, we generated quantitative predictions about how plant growth, disease severity, and infection prevalence in the flax rust system might change in future climates. These results can serve as a first approximation for how the dynamics of other fungal diseases of plants may change. More broadly, this effort represents an important bridge between the fields of infectious disease dynamics and plant pathology by introducing a modeling framework for making semi-mechanistic projections of plant infectious disease spread in future climates. Developing a more complete understanding of plant disease in a changing world will require further integrations of approaches and ideas from each field.

## Methods

### Data collection

#### Study sites

We selected four study sites in the Upper Gunnison Basin in Colorado, USA. As we intended for these sites to capture a large amount of variation in environmental conditions, the primary criteria we used for site selection was elevation. Mirroring patterns of mountain weather observed worldwide, elevation is correlated with a multitude of environmental factors in the Rocky Mountains. Predominant trends include a negative correlation between altitude and temperature, a positive correlation between altitude and precipitation^34^, and transitions in plant community composition (from ‘mountain shrub’ to ‘montane’ to ‘subalpine’ to ‘alpine’) with increasing altitude^35^.

Our sites span a large elevation gradient of approximately 1000 m that captures significant variation in environmental conditions. These sites should not be interpreted as a direct ‘elevation for climate substitution’, especially considering the significant changes in weather at each site that occur over the course of our observation periods, which ran from June to July. The lowest site, ‘cement creek’ (CC; approximately 38.82156° N, 106.86893° W), falls within a sage brush meadow on the boundary between the mountain shrub and montane vegetation zones at 2440 m elevation. The second lowest site, ‘bus turnaround’ (BT; approximately 38.97130° N, 106.99595° W), is situated in an open subalpine meadow at the base of Gothic Mountain at ~ 2940 m. The second highest site, ‘gothic mountain’ (GM; approximately 38.97969° N, 107.01937° W), is also in the subalpine zone on a steep hillside within a clearing in an evergreen forest on the lower slopes of Gothic Mountain at 3220 m. The highest site, ‘high meadow’ (HM; approximately 38.96779° N, 107.02184° W), is on an exposed meadow at the upper fringes of the subalpine zone on the shoulder of Gothic Mountain at 3,410 m.

We began our observations at each site by mapping the flax population (see “Population mapping” sub-section below). This occurred on 6/15/2020 for CC, 6/17/2020 for BT, 6/16/2020 for GM, and 6/18/2020 for HM. We concluded all observations at CC on 7/27/2020, at BT on 7/29/2020, at GM on 7/28/2020, and at HM on 7/10/2020 (aside from spore trap collection which concluded on 7/21/2020).

#### Weather monitoring

We deployed environmental sensors to record longitudinal weather data at each of our sites. We recorded temperature, humidity, rainfall, and wind speed and direction (at ~ 1 m above ground) every five minutes (see Supplementary Text 1 for logger specifications). We used temperature and humidity data to calculate absolute humidity (Supplementary Text 2). Due to logistical constraints, rainfall and wind speed/direction loggers were deployed later than temperature and humidity loggers.

We extracted weather metrics for a given observation period from this data as follows: We calculated the mean, maximum, and minimum temperature as the mean, maximum, or minimum of temperature readings. Likewise, we calculated mean absolute humidity as the mean of absolute humidity records (calculated from temperature and relative humidity every five minutes). Daily mean rainfall was calculated as of mean rainfall records multiplied by the number of 5 min increments in a day (i.e. 288).

#### Future climate data

To enable forecasting of the effects of climate change on within and between host transmission processes, we compiled projected future weather data. We extracted downscaled climate and hydrological data generated using the Localized Constructed Analogs (LOCA) method and the CESM1(CAM5) model from the “Downscaled CMIP3 and CMIP5 Climate and Hydrology Projections” archive^36–40^. Specifically, we extracted values for daily mean temperature, relative humidity, and rainfall from the CESM1(CAM5) hydrology model, and values for daily maximum and minimum temperature from the CESM1(CAM5) climate model for both the RCP4.5 and RCP8.5 emissions scenarios^41^. Temporally, this data spanned June through July in 2020, 2030, 2040, 2045, 2050, 2060, and 2070. Spatially, this data represents projections for the area spanning (38.9375, 39.0) latitude and (− 107.0, − 106.9375) longitude. This area is the finest grained spatial extent including the BT site for which projections were available.

#### Population mapping

In order to uncover drivers of population level epidemiological dynamics, it is necessary to characterize the ‘landscape of susceptibility’ in the host population. For spatially structured infectious disease systems such as flax rust, this involves documenting not only the infection status of each individual and any likely covariates of contact rate or susceptibility (e.g. size), but also the spatial arrangement of individuals. To accomplish this, we established 10 by 20 m transects at each of our field sites. We recorded the coordinates of each transect corner and the compass bearings associated with the transect axes (so that wind direction could be translated to the transect coordinate system) using a Garmin GPS unit (part #: 010-01735-10). At the beginning of the field season, we mapped the location, height, and infection status (healthy or diseased) of each plant within each transect. Plants with heights less than five centimeters were denoted as seedlings. While other metrics of size such as stem count could be used to form a more complete picture of plant size, we chose to use height alone as a proxy for size as it was the only measure that we could feasibly record for the many hundreds of plants within each transect. At the CC site, we were unable to map all healthy plants in certain areas of the transect due to time constraints, but we did record data for all diseased plants in these regions.

#### Epidemiological surveys

After documenting the initial epidemiological conditions, we tracked the spatiotemporal spread of flax rust within each transect. Approximately once per week, we visually inspected each flax individual in the transect for signs of disease, recording the location of any newly infected plants so they could be matched to a previously uninfected plant. We also recorded the height of all newly infected plants. We conducted these epidemiological surveys at least seven times at each site. Plants identified as infected in the initial population mapping and in these subsequent epidemiological surveys were marked with flags to ensure that they would not be repeatedly recorded as newly infected. Although we did not map the entirety of the CC transect, we covered the entire 10 by 20 m area in all epidemiological surveys.

#### Diseased focal plants

Within each transect, we designated a subset of diseased plants as ‘focal diseased plants’ and used them to make detailed measurements of within-host disease spread processes. These processes included changes in infection intensity, which we measured at the plant scale, as well as pustule growth, which we measured at the sub-plant scale using changes in pustule area and as a proxy.

All initially infected plants were designated as diseased focal plants, and we continued to give this designation to newly diseased plants (except in some cases, diseased plants with height of approximately 5 cm or less, as these plants were too delicate to be manipulated) until we obtained at least 25 infected focal diseased plants per site. This occurred on 7/20/2020 at CC, on 7/1/2020 at BT, and on 7/9/2020 at GM and HM. We inserted metal tags into the ground at the base of diseased focal plants so they could be quickly and reliably identified. To facilitate longitudinal measurements of disease spread at sub-plant scales, we marked up to three infected stems on each focal diseased plant with pieces of colored flagging tape tied around the base of the stem. These stems were chosen haphazardly. On each of these stems (when possible), we marked the tip of one infected leaf with black ink. We preferentially marked leaves with one or a few pustules. Approximately once every three days, we recorded detailed measurements of plant height and within-host disease spread processes.

To measure pustule growth, we photographed each marked leaf with a millimeter ruler in frame using a Cannon EOS Rebel T7i DSLR camera fitted with a Canon EF-S 35 mm f/2.8 Macro IS STM lens and a Cannon MR-14EX II Macro Ring Lite. While achieving consistent photographic angles and lighting conditions is impossible in a field setting, we attempted to keep the leaf and the scale in the same plane while maintaining a perpendicular shooting angle. We also adjusted camera settings so that pustule boundaries could be clearly distinguished in images. We used ImageJ software^42^ to measure the maximum and minimum diameters of each photographed pustule, and calculated pustule area assuming pustules to be elliptical. To enable the size of individual pustules to be tracked across observations, each measured pustule in each image was labeled so that it could be re-identified in subsequent images based on its location on the leaf and position relative to other pustules. We omitted data for any pustule that could not be confidently identified or whose borders became indistinct from other pustules. We marked a new leaf when we were unable to find the previously marked leaf, when the leaf was accidentally removed during data collection, or when the condition of the leaf deteriorated significantly. We suspect that either the presence of ink or our handling of the plant contributed significantly to the rate at which leaf condition deteriorated, but nevertheless we were able to observe the growth of many pustules over many weeks.

To measure infection intensity, we first counted the number of infected stems. Next, for each marked stem, we recorded the length of infected tissue as the length of the stem segment spanning the lowest (relative to the ground) pustule to the highest pustule. Finally, we counted the number of pustules present on a haphazardly selected leaf in the middle of the region of infected tissue on each marked stem. We did not use the same leaf at each observation as the middle of the region of infected tissue changed due to disease spread. Using these measurements, we calculated an infection intensity metric as the product of the number of infected stems, the average length of infected tissue, and the average number of pustules present on a leaf in the middle of the region of infected tissue.

#### Healthy focal plants

As we wished to investigate patterns of growth in both healthy and infected plants, we designated certain uninfected plants as healthy focal plants, and measured their height whenever we measured the height of focal diseased plants. To control for potential effects of stem marking on the growth of diseased focal plants, we made these same markings on healthy focal plants. Healthy focal plants were selected haphazardly, and we added new healthy focal plants over time to keep the number of diseased and healthy focal plants approximately equal. At some sites, the number of healthy focal plants fell towards the end of the observation period as many healthy focal plants became infected. When this occurred, we stopped recording data for the plant, and in some cases designated it as a diseased focal plant.

#### Spore deposition

To measure the distribution of spore deposition from an infected plant, and how it relates to the infection intensity of the source plant and wind patterns, we deployed spore traps in arrays around a subset of focal diseased plants. We chose focal disease plants that were as removed as possible from other diseased plants to minimize the number of spores originating from other plants that would be caught in the spore traps. These spore traps consisted of a ~ 2 cm^2^ section of Scotch Permanent Clear Mounting Tape (part #: MT76272-5) affixed to plastic backing with double sided tape. These spore traps were secured into the ground with ~ 5 cm nails. We deployed these traps at distances of either {~ 5 cm} (traps at ~ 5 cm were placed as close to the plant as possible), {~ 5 and 25 cm}, {~ 5, 25, and 50 cm}, or {~ 5, 25, 50, and 100 cm} in each of the four directions corresponding to the axes of the transect in which they were located. The traps were left in the field for approximately one week, and then collected. The sections of mounting tape were transferred to microscope slides and sealed in place using clear packing tape. We then used a light microscope to count the number of spores present in a known area. We counted spores in an area of 0.8 cm^2^ for most spore traps that contained few or no spores, but we counted a minimum area of 0.1 cm^2^ for all spore traps, including those with greater than 10,000 spores per cm^2^. *Melampsora lini* spores were identified visually using a slide prepared with spores as a reference for spore size, shape, and color. The spores matched descriptions in the literature^43^. We did not observe any rust fungi other than *M. lini* in or around our transects, so we can be reasonably certain that our counts represent only *M. lini* spores from infected focal diseased plants.

### Statistical analyses

#### Plant growth and within-host disease spread

To infer how different weather factors affect plant growth, pustule growth, and changes in infection intensity, we fit generalized additive models^18^. We used longitudinal observations of plant height in healthy and diseased focal plants to fit the model of plant growth, and longitudinal observations of within-host disease spread at various scales in focal diseased plants to fit all other models. Because the observation periods associated with our measurements of these processes varied, we formatted the response variables in change-per-day units: For our analyses of plant growth, pustule growth, and infection intensity progression we used change in plant height per day, change in pustule area per day, and change in infection intensity per day respectively as the response variables.

To infer the effects of weather variables, we included smooth terms for these factors as predictors in each model. For each observation of change in plant height, pustule area, or infection intensity, we determined the start and end timepoints of the observation period to extract the corresponding weather data. For observations of plant height, we used 12:00:00 on the day of the observation as the start/end timepoints of the observation period. For observations of pustule area, we used photograph timestamps as the start and end timepoints. For observations of infection intensity, we used the average of the timestamps of photographs taken of pustules on that plant’s leaves as the start and/or end timepoints if such photographs were taken, because all data for an individual diseased focal plant was generally collected at once. If such photographs were not taken, we used the mean timestamp of all photographs taken at that plant’s site on the observation date as the start and/or end timepoint. To focus our analysis on fine-grained relationships between weather and within-host disease spread, we discarded data corresponding to observation windows of eight or more days. According to this criterion, 13 of 609 data points were discarded in the analysis of plant growth, 216 of 3535 data points were discarded in the analysis of pustule growth, and 25 of 338 data points were discarded in the analysis of infection intensity progression. Using the start and end timepoints of the observation period as bounds, we extracted the following variables from the weather data: temperature (mean, maximum, and minimum), absolute humidity, and mean daily rainfall.

To account for the nested nature of our data, we included smooth terms for study site and plant identity as random effects (accomplished by setting bs = ”re” in the s() function in mgcv) in each model. In the case of the analyses of pustule growth, plant identity refers to the plant on which the pustules were observed. We also included a term in each model that accounted for the value of the previous observation to capture any allometric effects. In the model of change in plant height per day, this term was a full tensor smooth product of last observed height and infection intensity. In the model of change in pustule area per day, this term was a smooth of last observed pustule area. In the model of change in infection intensity per day, this term was a full tensor smooth product of the base 10 logarithm of the last observed infection intensity and last observed maximum plant height.

We fit these models using the gam() function in the mgcv R package and the restricted maximum likelihood parameter estimation method^18,44^. We used gaussian response distributions with identity link functions. The dimensionality of all basis functions was set to the default value determined by mgcv. The one exception to this involved the marginal basis for log_10_ infection intensity (a part of the tensor term) in the model of change in infection intensity per day. The dimensionality of this marginal basis was increased to 15 after model diagnostics indicated that the default basis dimension was insufficient. To implement variable selection so that insignificant predictor terms would be effectively removed from the models, we used the double penalty approach of Mara and Wood^45^ (via the ‘select = TRUE’ option in the mgcv gam() function).

The coefficients of the fit models can be used to infer the effects of weather factors on plant growth and within host disease spread. To translate these inferences into predictions about how climate change might affect plant growth and within-host disease spread, we simulated trajectories of plant growth, pustule area, and infection intensity under various climate conditions. For each class of simulation, we started by defining a hypothetical starting state. For simulations of plant growth, this was a 10 cm tall uninfected plant (the plant was assumed to remain uninfected for the entire simulation). For simulations of pustule growth, this was a pustule with area 0.1 cm^2^. For simulations of infection intensity, this was a 25 cm tall plant with infection intensity 1.0. Next, we simulated 100 trajectories from the model posteriors using observed weather data for each site and a step size of seven days. In these simulations, we restricted plant height, pustule area, and infection intensity to remain greater or equal to 5 cm, 0 mm^2^, and 0.1, respectively. We included the random effect of site (but not plant identity) when making these predictions. After confirming that these simulation results qualitatively recapitulated observed patterns, we simulated sets of 100 trajectories for future climate data sets. We again excluded the random effect of plant identity when making these projections. We used the same set of 100 random number seeds for each set of 100 trajectory simulations.

While performing these simulations, we extracted weather variables as follows: When extracting weather variables from observed data sets, we defined the observation period as 00:00:00 on the first day in a step to 00:00:00 on the last day in a step (e.g. midnight on July 1st to midnight on July 8th). We extracted weather metrics as described in the “Weather data” subsection of the “Data collection” section above. Applying these same methods to the projected weather data is not possible, as these data sets only give only daily mean values. Instead, we calculated mean temperature as the mean of daily mean temperature values, maximum temperature as the maximum of daily maximum temperature values, and minimum temperature as the minimum of daily minimum temperature values. Similarly, we used daily mean temperature and relative humidity to calculate daily mean absolute humidity, and calculated mean absolute humidity as the mean of these values. We applied this same procedure to calculate mean rainfall.

#### Spore dispersal

To infer how spore deposition is related to source plant infection intensity, wind speed, and wind direction, we fit a tilted gaussian plume model^22^ (TGPM) to our spore deposition data. The equation specifying the TGPM is as follows:

$$\begin{aligned} & d\left(I,H,s,X,Y\right)=\frac{I k {W}_{s}}{2 \pi s {\sigma }^{2} }{e}^{\frac{{-Y}^{2}}{2{\sigma }^{2}}-\frac{{(H-\frac{{W}_{s }X}{s})}^{2}}{2{\sigma }^{2}}}  \\ & for \quad X>0 0 \\ &  for \quad X\le 0.\end{aligned}$$

This equation describes the concentration of spores deposited at a given point ($d$) as a function of wind speed ($s$), the infection intensity of the source plant $(I)$, a constant relating $I$ to the source concentration of spores $(k)$, and the coordinates of the point (X,Y), relative to the source. The coordinate system has the source at the origin, the X-axis parallel to the wind direction (with wind flowing in a positive direction), the Y-axis perpendicular to the X-axis on the plane defined by the ground (assumed to be flat), and the vertical Z-axis orthogonal to the X and Y axes. The shape of the three dimensional spore plum emanating from the source is defined by $s$, along with constants specifying the falling velocity of spores $({W}_{s})$, the height of the source ($H$), and the standard deviation of spore dispersion (${\sigma }^{2}$) in the horizontal and vertical directions. Following Levine and Okubu^22^, we calculate ${\sigma }^{2}$ as a function of a diffusion coefficient, $A$, assuming that the variance in spore distribution increases linearly with time. We also assume that spores diffuse along the Y and Z axes at equal rates.

$${\sigma }^{2}=\frac{2AX}{s}.$$

We fit the parameters $k, {W}_{s},$ and $\sigma$ by minimizing the sum of squared differences between model predictions and the spore deposition data we collected from the spore traps arranged around diseased focal plants. We assumed that all spores deposited on a spore trap originated from the associated diseased focal plant, and that spore deposition occurred between noon on the day of deployment and ceased at noon two days post-deployment as the spore traps lost their adhesive properties and became saturated with dust. When predicting spore deposition using the TGPM, we set $I$ equal to the infection intensity of the source diseased focal plant on the day of spore trap deployment and $H$ equal to half of the maximum height of the source diseased focal plant at the day of spore trap deployment. As wind speed and direction were recorded every 5 min, we calculated total spore deposition as the sum of predicted deposition across five minute time windows. In each individual estimate, we set $s$ equal to wind speed and calculated $X$ and $Y$ from wind direction and the location of the spore trap relative to the focal diseased plant (see Supplementary Text 3).

#### Transmission

Using the fitted TGPM we next sought to connect patterns of spore dispersal to transmission. The first step in this process was to align data on the spatiotemporal spread of disease with the locations of all healthy and infected plants (including non-focal plants) within each transect so that the relative locations of these could be determined (see Supplementary Text 4). Next, we built a dataset (‘corrected plant height data’) describing the height of all plants on the dates of epidemiological surveys. We preferentially used actual measurements from the initial population survey, epidemiological surveys, or focal plant measurements where they existed. For the majority of plants, height was not recorded on the date of a given epidemiological survey. In these cases, we fore- or hind-casted height from a previous or future record of plant height (whichever was closer) using the fitted generalized additive model of plant growth and observed weather data spanning the period between the date of interest and the closest record. We included the random effect of study site when making predictions, but not the random effect of plant identity as we wished to forecast heights of both focal and non-focal plants and only focal plant data was used to fit the model of plant growth.

After completing these steps, we next built a ‘transmission data set’ connecting weather conditions, predicted spore deposition, plant height, and infection occurrence across the time windows between each epidemiological survey. We included separate entries for each healthy plant (focal and non-focal) during each time window. We limited the scope of this data to the periods for each transect in which all infected plants (that were not seedlings) were tracked as diseased focal plants. Using noon on the date of one epidemiological survey as the beginning of the time window, and noon on the date of the next epidemiological survey as the end of the time window, we extracted the same weather variables as described in “Plant growth and within-host disease spread”. Plant height values were extracted from the ‘corrected plant height’ data set. We determined that a plant became infected if a plant with identical coordinates (after data alignment) was recorded as newly infected in the epidemiological survey that took place at the end of the observation period. Total spore deposition for each healthy plant was calculated as the sum of predicted spore deposition originating from each of the plants in the same transect that was infected at the beginning of the time window. To predict the spore deposition experienced by a healthy plant that originated from an individual diseased plant, we used the TGMP$.$ We extracted the height of the diseased plant at the beginning of the time window and set $H$ to half of this value. Likewise, we extracted the infection intensity of the diseased plant at the beginning of the time window, and set I equal to this value. For diseased seedlings that were not tracked as diseased focal plants, we set I = 0.1, as most were observed to be lightly infected. There were 14 instances of missing infection intensity data for a diseased focal plant on a certain date. In these cases, we fore- or hind-casted infection intensity for that plant using the generalized additive model of infection intensity progression fit in “Plant growth and within-host disease spread” (while including the random effect for study site, but not for plant identity)*,* the closest observation of infection intensity for that plant, and the weather data spanning the time period from the missing observation to the closest measurement. We set the values of $k, {W}_{s},$ and $\sigma$ to those fit in the “spore deposition” section above. As wind speed and direction were recorded every five minutes, we summed spore deposition over 5 min windows that matched the resolution of the wind speed and direction data. For each of these windows, we predicted spore deposition after setting the values of X and Y based on the relative location of the healthy and diseased plants and the direction of the wind (see Supplementary Information).

Using this data set, we modeled infection outcomes in a generalized additive model framework. We assumed a binomial response distribution, coding the outcome of an infection as 1 and the outcome of no infection as 0. To account for variation in the duration of observation period, we used a complementary log–log link function, and included the log time as an offset in the model^46^. We included smooth terms for all weather variables as predictors, along with smooth terms study site and plant identity as random effects (accomplished by setting bs = ”re” in the s() function in mgcv). To infer the connection between spore deposition and infection, we also included a full tensor smooth product between the base 10 logarithm of mean total spore deposition per day and maximum plant height. When fitting the tensor, we fixed the smoothing parameter for the marginal smooth for log_10_ total spore deposition to $1\times {10}^{10}$. This effectively sets the shape of this marginal smooth to be linear, preventing any overfitting involving non-monotonic effects of spore deposition. Height was included as a factor in the tensor for two reasons. First, we calculated spore deposition for a point. In reality, the spore deposition experienced by a plant depends on the size of the ‘target’ that it presents. As such, larger plants are presumably challenged by a greater number of spores than a smaller plant for the same level of predicted spore deposition. Secondly, plant size could be correlated with quantitative resistance, because leaf age can influence pustule development^20^. To achieve computational feasibility, we fit this model using the bam() function in the mgcv R package and the fast restricted maximum likelihood parameter estimation method (“fREML”). We again implemented variable selection so that insignificant predictor terms would be effectively removed from the models via the double penalty approach.

To infer the effects of climate change and to illustrate the relationships between spore deposition, plant height, and infection risk, we predicted odds of infection using the estimated parameters of the fitted GAM (Fig. 5; Supplementary Fig. S14) and various weather data sets. In all of these projections, we included the random effect of site but excluded the random effect of plant identity.

### Epidemiological model

#### Model description

To predict how the population level epidemiological dynamics of flax-rust might be affected in various climate change scenarios, we constructed a spatio-temporal epidemic model. This model tracks the infection status, infection intensity, and height of plants over time as infection spreads within the modeled population. There are three components required to simulate the model: (1) initial conditions describing the locations and starting states of all plants, (2) weather data, and (3) models of spore dispersal, transmission, infection intensity progression, and plant growth.

The initial conditions for individual simulations of the epidemic model were in all cases constructed to represent either the BT or GM study populations on the date of an early epidemiological survey. For BT, this date was chosen as the first which had complete weather data (spanning 00:000:00 to 24:00:00), which was 6/24/2020. For GM, this date was set to 7/2/2020. Weather data was available for earlier dates, but a sharp increase in prevalence occurred during the prior week, perhaps due to prior missed observations of new infections. The model consistently underestimated observed prevalence when initialized prior to this increase in prevalence. We did not use the HM study population to initialize the model as no epidemiological surveys occurred on a date with complete weather data. Likewise, we do not use the CC study population to initialize the model because not all regions of the transect were mapped (see “Population mapping” sub-section in “Data collection” above).

The construction of initial conditions to represent a given site proceeded as follows. The locations of all plants were taken from the ‘corrected plant height’ data set. Plants observed to be infected on the date of the first epidemiological survey were initialized as infected, and all other plants were initialized as uninfected. Following the first epidemiological survey, all infected plants (except for several that were approximately 5 cm or less in height) were designated diseased focal plants and their infection intensity was recorded. We used these measurements as the initial infection intensities of infected plants. We fore- or hind-casted infection intensities for any missing records using the method described in the transmission sub-section of the statistical analyses section above. Plant heights were taken from the ‘corrected plant height’ data set.

The weather data component of the model is used to predict spore dispersal, transmission, infection intensity progression, and plant growth in conjunction with the fitted statistical models of these processes. As such, it needs to describe mean weather conditions over the period spanning a simulation step. Additionally, because the TGPM is calibrated to make predictions over 5 min intervals, the weather data must also describe average wind speed and direction over five minute windows.

We used the fitted statistical model relating weather to the processes spore dispersal (TGPM), transmission, infection intensity progression, and plant growth to simulate these processes in the epidemiological model. These models contain random effects for both site and plant identity. The random effect of plant identity was excluded when making predictions about infection intensity progression and plant growth as random effects were only fit for focal plants. We included random effects of plant identity when making predictions using the transmission model because random effects were fit for all plants in the modeled populations.

Using these components, the model simulates changes in infection status, infection intensity and plant height in time steps of 7 days. The simulation for each time step proceeds as follows:

1. First, the model predicts the number of spores deposited at the location of each healthy plant over the course of the entire time step. As all infected plants are assumed to be sources of spore deposition, the model predicts total spore deposition as the sum of spore deposition originating from each infected plant. The spore deposition originating from a single infected plant is calculated as the sum of predictions over 5 min windows spanning the time step. Individual predictions were generated using the TGPM with I is set to the infection intensity of the source plant at the beginning of the time step, H set to 0.5 times the height of the source plant at the beginning of the time step, s set to average wind speed, and X and Y set according to locations of the source and target plants and wind direction (see Supplementary Information). The parameters $k, {W}_{s},$ and $\sigma$ were set to the values fit to spore trap data.
2. Next, for each healthy plant, the model infers the probability of that plant becoming infected from predicted spore deposition at that plant’s location and mean weather metrics using the transmission model. Following this, a random number in [0,1] is drawn, and if it is equal or lower to the probability of infection that plant becomes infected. All newly infected plants are set to have an infection intensity of 1.0.
3. After simulating the transmission process, the model next simulates changes in infection intensity. For each plant, we simulated a prediction of the change in infection intensity per day by drawing randomly from the posterior of the infection intensity progression model. We used the infection intensity and height of that plant at the beginning of the time step along with mean weather metrics spanning the time step as the model inputs. We multiplied the predicted change in infection intensity per day by the length of the time step and added it to the plant’s infection intensity at the beginning of the time step to calculate the new infection intensity of the plant.
4. Finally, we used a similar process to simulate change in plant height. For each plant, we simulated a prediction of the change in height per day by drawing randomly from the posterior of the plant growth model. Again, we used the height of that plant at the beginning of the time step along with mean weather metrics spanning the time step as model inputs. To calculate the new height of the plant, we multiplied the predicted change in height per day by the length of the time step and added this number to the plant’s height at the beginning of the time step.

There are two sources of stochasticity within the epidemiological model. The first of these sources pertains to the probability of infection. Although the predicted probability of infection for a certain combination of predictor values is always the same, we compare this probability to a random number to determine if infection occurs. The second source of stochasticity pertains to predicted changes in infection intensity and plant height. We predict rates of infection intensity change and plant growth by drawing randomly from the posterior distributions of the corresponding fitted models. These sources of stochasticity allow us to understand the variation in epidemiological model predictions via repeated simulation.

#### Simulation

To validate the epidemiological model, we simulated 10 epidemiological trajectories for the BT and GM sites and compared the results to our observations of flax-rust spread to ensure a qualitative match. For each of these sites, we initialized the model using data corresponding to the site as described above and used observed weather (from the same site) data to run the simulation. When extracting weather variables from observed weather data sets, we defined the observation period as 00:00:00 on the first day in a step to 00:00:00 on the last day in a step (e.g. noon on July 1st to noon on July 8th). We included the random effects of site and plant identity when making predictions from the transmission model, and the random effects of site when making predictions from the infection intensity and plant growth models.

To infer the effects of climate change of flax-rust epidemiology, we again initialized the model using data corresponding to the for BT and GM sites, and then simulated 10 epidemiological trajectories for each set of weather data (RCP4.5 and 8.5 emissions scenarios and the years 2020, 2030, 2040, 2045, 2050, 2060 and 2070). We extracted weather metrics as described in the “Weather data” subsection of the “Data collection” section above. We used observed records of wind speed and direction to simulate spore deposition using the TGPM as the projected data sets lack these measures. As before we included the random effects of site and plant identity when making predictions from the transmission model, and the random effects of site when making predictions from the infection intensity and plant growth models. For each set of 10 model simulations corresponding to a unique site and weather data set combination, we used the same 10 random number seeds.

## Supplementary Information

Supplementary Information.

## Data Availability

All data and code used in our analyses is included as supplementary information.
