# Supplementary material for: Predicting the effects of climate change on the cross-scale epidemiological dynamics of a fungal plant pathogen
Source: Sci Rep. 2022 Sep 1;12:14823. doi: 10.1038/s41598-022-18851-z (PMC9437057; doi:10.1038/s41598-022-18851-z)
Supplement: Supplementary file 1 — Supplementary Information. [file 41598_2022_18851_MOESM1_ESM.docx]

**Supplementary Information**

Supplementary Text 1: Environmental condition monitoring equipment

We recorded temperature and humidity using HOBO Temperature/RH data loggers (part #: MX2301A) enclosed in solar radiation shields. These loggers were mounted approximately 30cm above ground level on wooden stakes. We recorded rainfall, wind speed, and wind direction using sensors connected to HOBO Micro Stations (part # H21-USB). Rainfall was measured using Davis (0.2 mm) Rain Gauge Smart Sensors (part # S-RGF-M0002). Wind speed and direction were measured using Davis Wind Speed and Direction Smart Sensors (part #: S-WCF-M003).

Supplementary Text 2: Absolute humidity calculation

To calculate absolute humidity (in terms of g H_2_O per cubic meter of air) from our observations of temperature and humidity, we began with the ideal gas law where P is pressure, V is volume, n is the number of gas molecules in mols, R is the universal gas constant, and T is the temperature in Celsius.

$$\begin{aligned} PV=nR\left( T+273.15 \right)\#\left( s1 \right) \end{aligned}$$

By setting $V=1$, substituting in the value of R, and rearranging variables, we can solve for $n$:

$$\begin{aligned} n=\frac{P}{0.08314\left( T+273.15 \right)}\#\left( s2 \right) \end{aligned}$$

In order for $n$ to represent the number of mols of water present in the gas, we need to substitute the partial vapor pressure of water, $p_{w}$, for P. We calculate the partial pressure of water by multiplying that saturation vapor pressure of water, $p_{ws}$, given by Teten’s formula^1^ by the relative humidity, $\phi$, expressed as a percentage:

$$\begin{aligned} p_{ws}=6.112 e^{\frac{17.67*T}{T+243.5}}\#\left( s3 \right) \end{aligned}$$

$$\begin{aligned} p_{w}=p_{ws}\frac{\phi}{100}\#\left( s4 \right) \end{aligned}$$

By substituting s3 and s4 into s2, we obtain the number of mols of water in 1 cubic meter of air as:

$$\begin{aligned} n=\frac{6.112 e^{\frac{17.67*T}{T+243.5}}\phi}{0.08314 \left( T+273.15 \right) 100}\#\left( s5 \right) \end{aligned}$$

By multiplying the right side of this expression by the molecular weight of water, 18.02g/mol, and simplifying, we finish our derivation of absolute humidity as a function of temperature and relative humidity:

$$\begin{aligned} absolute humidity \left( g\frac{H_{2}0}{m^{3}air} \right)=\frac{13.24732 e^{\frac{17.67*T}{T+243.5}}\phi}{\left( T+273.15 \right)}\#\left( s6 \right) \end{aligned}$$

Supplementary Text 3: Calculating values of $X$ and $Y$ in the tilted gaussian plume model

To estimate spore deposition using the TGPM it is necessary to find the values of X and Y from wind direction and the coordinates of the source plant and target within the transect. Figure S11 gives a visual representation of this problem. This is accomplished as follows:

First, we define a coordinate system such that the corners of the transect are located at $\left( 0,0 \right),\left( 10,0 \right),\left( 0,20 \right),(10,20)$. In the raw wind data, a wind direction ($\theta$) of $0^{\circ}$ or ${360}^{\circ}$ corresponded to north. We applied a site specific correction so that a corrected wind direction ($\theta^{*})$ of $0^{\circ}$ or ${360}^{\circ}$ would point in the same direction as $\langle0,1\rangle$:

$$\begin{aligned} \theta^{*}=\theta-{332}^{\circ} for site CC\#\left( s7a \right) \end{aligned}$$

$$\begin{aligned} \theta^{*}=\theta-{75}^{\circ} for site BT\#\left( s7b \right) \end{aligned}$$

$$\begin{aligned} \theta^{*}=\theta-{309}^{\circ} for site GM\#\left( s7c \right) \end{aligned}$$

$$\begin{aligned} \theta^{*}=\theta-{40}^{\circ} for site HM\#\left( s7d \right) \end{aligned}$$

Let $(x_{S},y_{S})$ and $(x_{T},y_{T})$ be the coordinates of the source plant and target respectively. Using the direction of the wind, we can define a point $(x_{W},y_{W}$) such that the line passing through $(x_{S},y_{S})$ and $(x_{W},y_{W}$) points in the direction of the wind:

$$\begin{aligned} x_{W}=x_{S}+\sin\left( \theta^{*} \right)\#\left( s8a \right) \end{aligned}$$

$$\begin{aligned} y_{W}=y_{S}+\cos\left( \theta^{*} \right)\#\left( s8b \right) \end{aligned}$$

For convenience, we name the line passing through $(x_{S},y_{S})$ and $(x_{W},y_{W}$) $W$. We wish to find the point $(x_{p},y_{p})$ on $W$ that is closest to $\left( x_{T},y_{T} \right)$, so that X can be calculated as the length of the segment $\bar{X}$connecting $(x_{S},y_{S})$ and $(x_{p},y_{p})$, and Y can be calculated of the length of the segment $\bar{Y}$ connecting$(x_{P},y_{P})$ and $(x_{T},y_{T})$. The coordinates of points on W can be written as

$$\begin{aligned} \left( x_{S}+x_{W} c , y_{S}+y_{W} c \right)\#\left( s9 \right) \end{aligned}$$

where $c$ is a scalar. We can define $D$, the squared distance between a point on W and $\left( x_{T},y_{T} \right)$, as a function of $c$:

$$\begin{aligned} D\left( c \right)=\left( x_{T}-\left( x_{S}+\left( x_{W}-x_{S} \right)c \right) \right)^{2}+{(y}_{T}-{\left( y_{S}+\left( y_{W}-y_{S} \right)c \right))}^{2}\#\left( s10 \right) \end{aligned}$$

We next find the derivative of $D(c)$:

$$\begin{aligned} D^{'}(c)=2\left( x_{T}-\left( x_{S}+\left( x_{W}-x_{S} \right)c \right) \right)\left( x_{W}-x_{S} \right)+ 2\left( y_{T}-\left( y_{S}+\left( y_{W}-y_{S} \right)c \right) \right)\left( y_{W}-y_{S} \right)\#\left( s11 \right) \end{aligned}$$

We can find the value of c that can be used to calculate $(x_{p},y_{p})$ by setting $D'\left( c \right)=0$ and solving:

$$\begin{aligned} c=\frac{\left( x_{T}-x_{S} \right)\left( x_{W}-x_{S} \right)+\left( y_{T}-y_{S} \right)\left( y_{W}-y_{S} \right)}{\left( x_{W}-x_{S} \right)^{2}+\left( y_{W}-y_{S} \right)^{2}}\#\left( s12 \right) \end{aligned}$$

We can then use this value of $c$ to construct the following equations for $x_{p}$ and $y_{p}$:

$$\begin{aligned} x_{P}=x_{S}+x_{W} \left( \frac{\left( x_{T}-x_{S} \right)\left( x_{W}-x_{S} \right)+\left( y_{T}-y_{S} \right)\left( y_{W}-y_{S} \right)}{\left( x_{W}-x_{S} \right)^{2}+\left( y_{W}-y_{S} \right)^{2}} \right)\#\left( s13a \right) \end{aligned}$$

$$\begin{aligned} y_{P}=y_{S}+y_{W} \left( \frac{\left( x_{T}-x_{S} \right)\left( x_{W}-x_{S} \right)+\left( y_{T}-y_{S} \right)\left( y_{W}-y_{S} \right)}{\left( x_{W}-x_{S} \right)^{2}+\left( y_{W}-y_{S} \right)^{2}} \right)\#\left( s13b \right) \end{aligned}$$

The absolute values of X and Y can then be solved for as:

$$\begin{aligned} \left| X \right|={{((x_{P}-x_{S})}^{2}+\left( y_{P}-y_{S} \right)^{2})}^{\frac{1}{2}}\#\left( s14a \right) \end{aligned}$$

$$\begin{aligned} \left| Y \right|={{((x_{P}-x_{T})}^{2}+\left( y_{P}-y_{T} \right)^{2})}^{\frac{1}{2}}\#\left( s14b \right) \end{aligned}$$

Because the shape of the spore distribution predicted by the TGPM is always symmetrical, we can set $Y=\left| Y \right|$. However, spore deposition only occurs for $X>0$, and as such it is necessary to determine the sign of $X$. We take advantage of the fact that the points $(x_{S},y_{S})$, $(x_{W},y_{W}$), and $\left( x_{p},y_{p} \right)$ all fall on W, and use the following set of rules to determine the sign of $X$:

$$\begin{aligned} \#s\left( 15 \right) \end{aligned}$$

- $forx_{W}>x_{S}$
  - $for x_{P}>x_{S}, X>0$
  - $for x_{P}<x_{S},X<0$
- $forx_{W}<x_{S}$
  - $for x_{P}>x_{S}, X<0$
  - $for x_{P}<x_{S},X>0$
- $for x_{W}=x_{S}$
  - - $for y_{W}>y_{S}$
    - $for y_{P}>y_{S}, X>0$
    - $for y_{P}<y_{S}, X<0$
    - $for y_{W}<y_{S}$
    - $for y_{P}>y_{S}, X<0$
    - $for y_{P}<y_{S}, X>0$

Supplementary Text 4: Plant location alignment

We corrected the coordinates of newly infected plants (recorded in ‘epidemiological surveillance’) so that they could be precisely location matched to previously uninfected plants (whose locations were recorded in ‘population mapping’). We expected slight differences between coordinates recorded during ‘epidemiological surveillance’ and ‘population mapping’ due to measurement error. Many newly infected plants were marked with plant tags, either because they were healthy focal plants, or because they were being tracked as a part of a concurrent demography study. We updated the coordinates of newly infected tagged plants to match those recorded for those same plants during ‘population mapping’. For newly infected untagged plants, this precise matching process was not possible. Instead, we matched these plants to the closest untagged, previously uninfected, and unmatched plant within 25cm, and updated their coordinates accordingly. If the newly infected plant was not able to be matched, then we did not correct its coordinates, but rather retroactively added a healthy plant with those same coordinates to the plant location dataset generated via ‘population mapping’. We added these records so that all infected plants would appear in data used to validate the epidemiological model, but we excluded these added records from our statistical analysis in the *‘Transmission’* section to avoid biasing our dataset towards infection events.

**Supplementary figures**


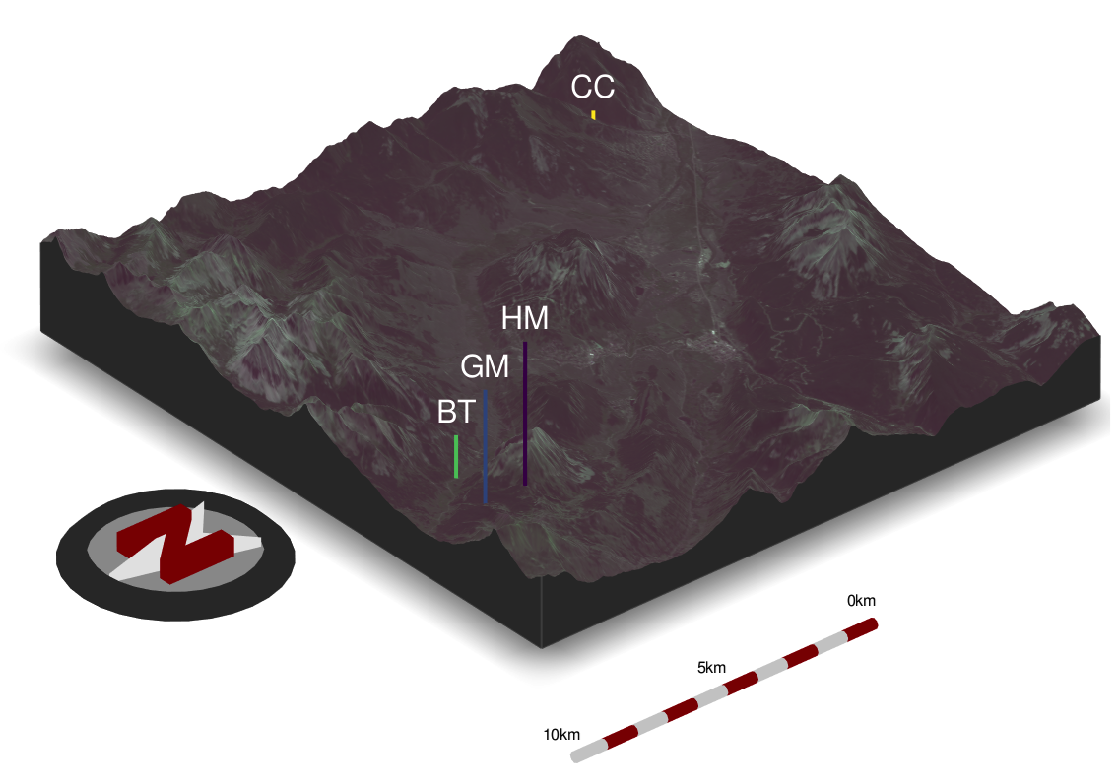


**Supplementary Figure 1: Study site map**

Vertical lines show the relative locations of the ‘Cement Creek’ (CC), ‘Bus Turnaround’ (BT), ‘Gothic Mountain’ (GM), and ‘High Meadow’ (HM) study sites. Elevation is not to scale. This figure was generated using the *rayshader* R package^2^ and Landsat-8 satellite imagery (courtesy of the U.S. Geological Survey).

**
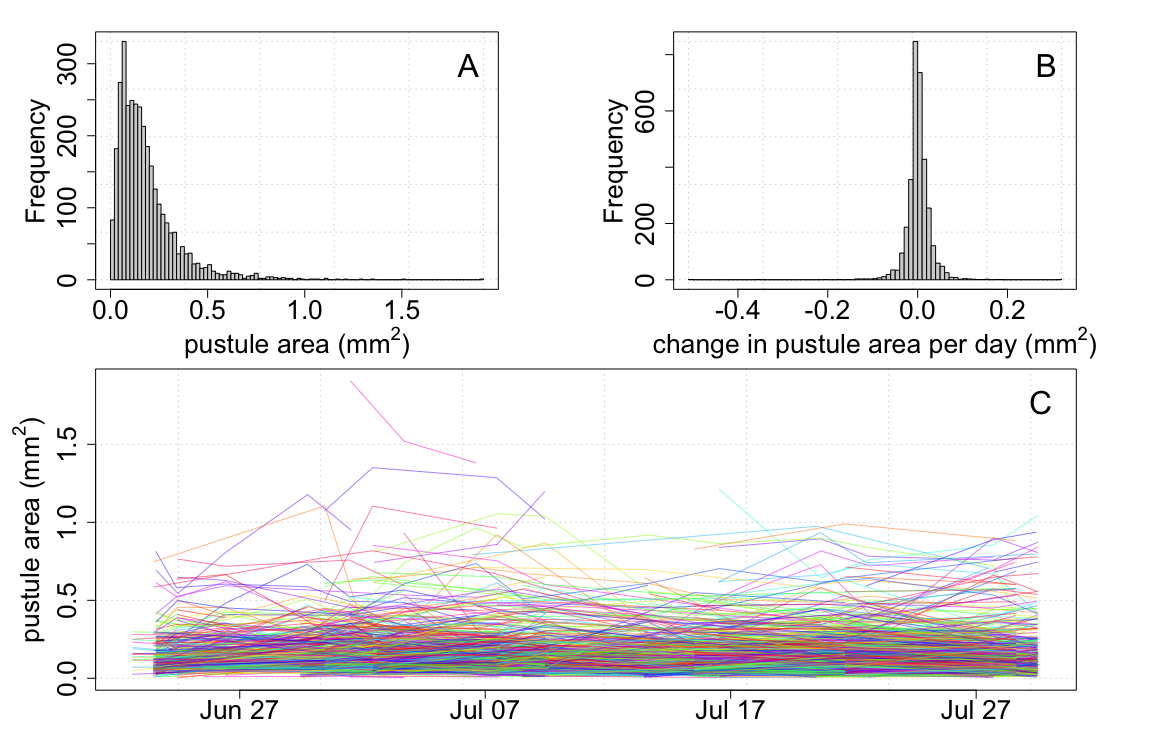
**

**Supplementary Figure 2: Pustule area data**

Panel A shows the distribution of pustule area measurements. Panel B shows the distribution of change in pustule area per day, the response variable in the GAM fit to infer climate effects on the replication rate of *M. lini*. Panel C shows the observed trajectories of pustule area, with each line representing an individual pustule.


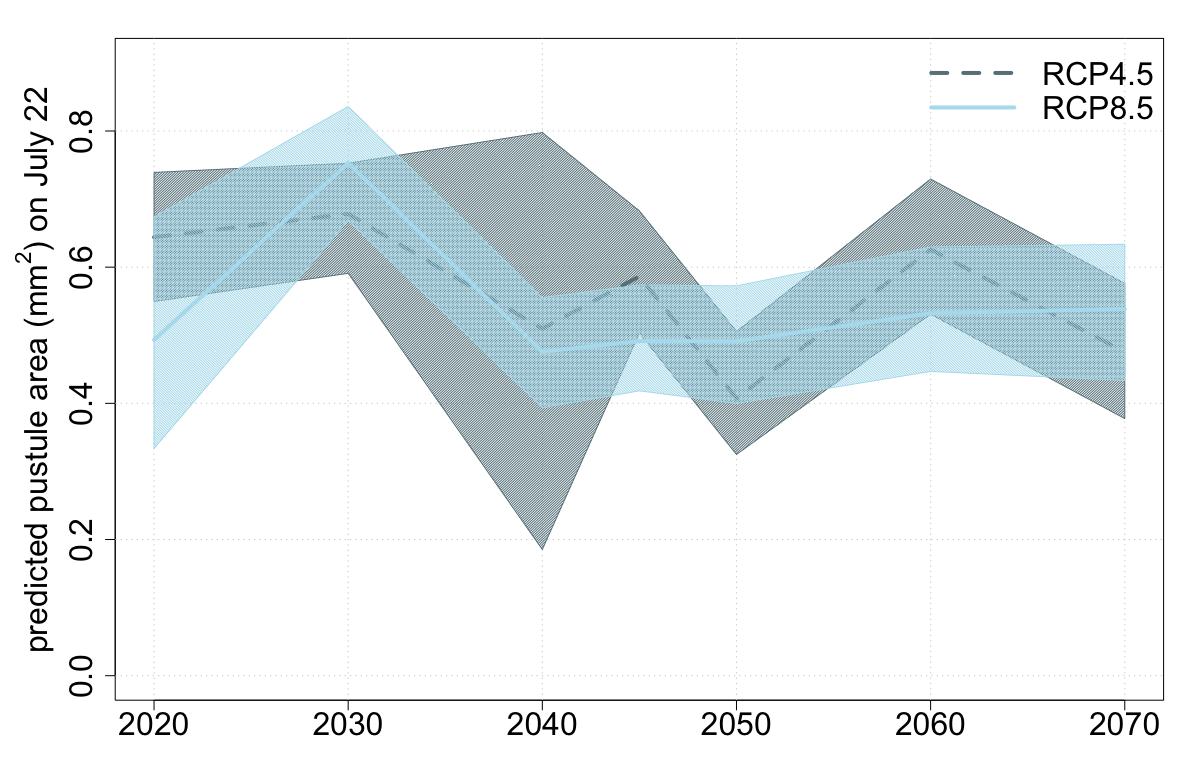


**Supplementary Figure 3: Patterns of end-of-simulation pustule area across future climate scenarios**

We simulated 100 trajectories of pustule growth (for a pustule starting with area 0.1 mm^2^) at the GM site for each of 12 future weather data sets encompassing the RCP 4.5 and 8.5 emissions scenarios and the years 2020, 2030, 2040, 2045, 2050, 2060, and 2070. Lines show the mean predicted pustule area at the end of the simulation for each year and each emissions scenario. Shaded regions span the 10% to 90% quantiles of predictions.

**
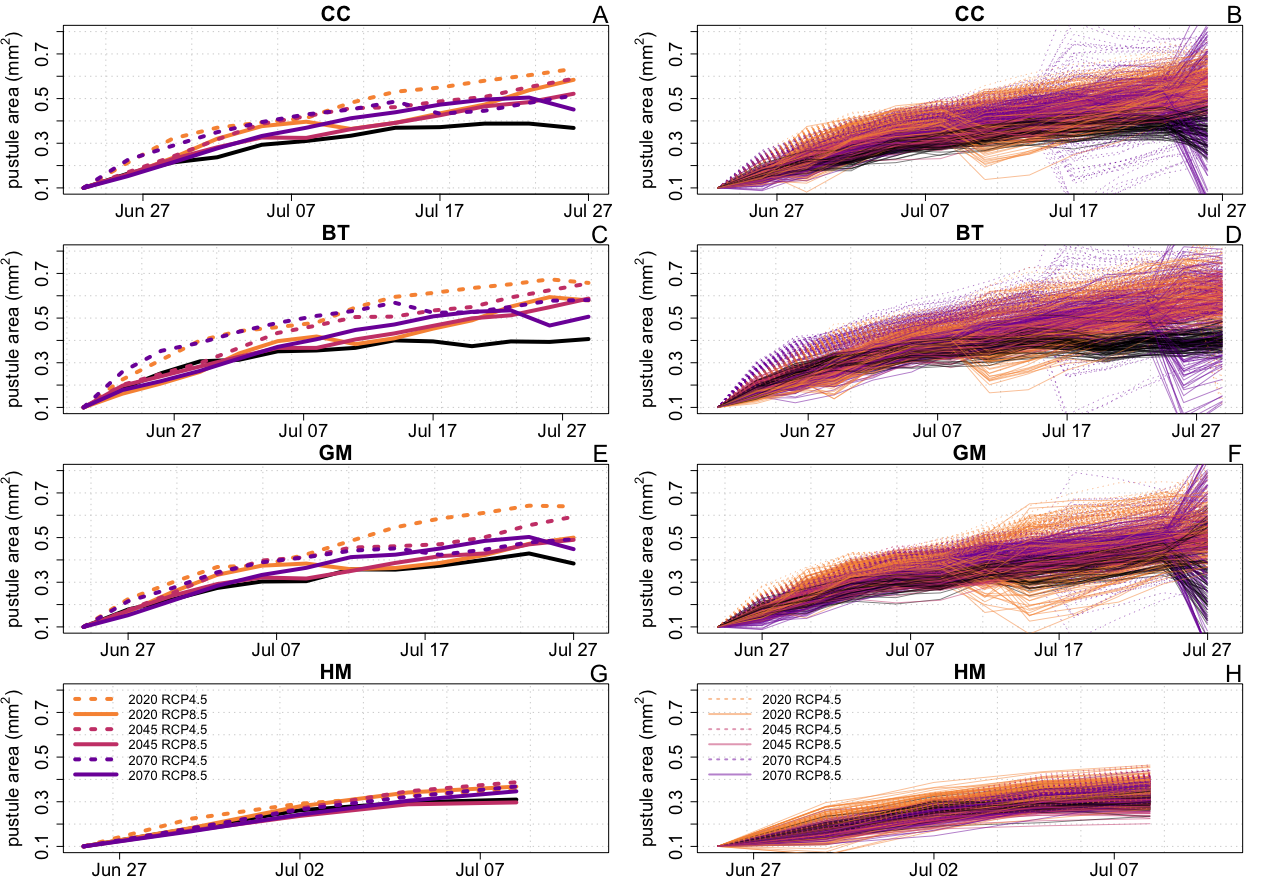
**

**Supplementary Figure 4: Simulated effects of future climate on pustule growth across study sites**

Using the fitted GAM model, we 100 simulated trajectories of pustule growth (for a pustule starting with area 0.1 mm^2^) at the four study sites for each of six future weather data sets. Lines in panels A, C, E, and G show the mean of the trajectories simulated for each weather condition. Lines in panels B,D,F, and H show individual trajectories. Panels A and B correspond to the CC site, panels C and D correspond to the BT site, panels E and F correspond to the GM site (panel E is identical to Fig. 2J), and panels G and H correspond to the HM site.

**
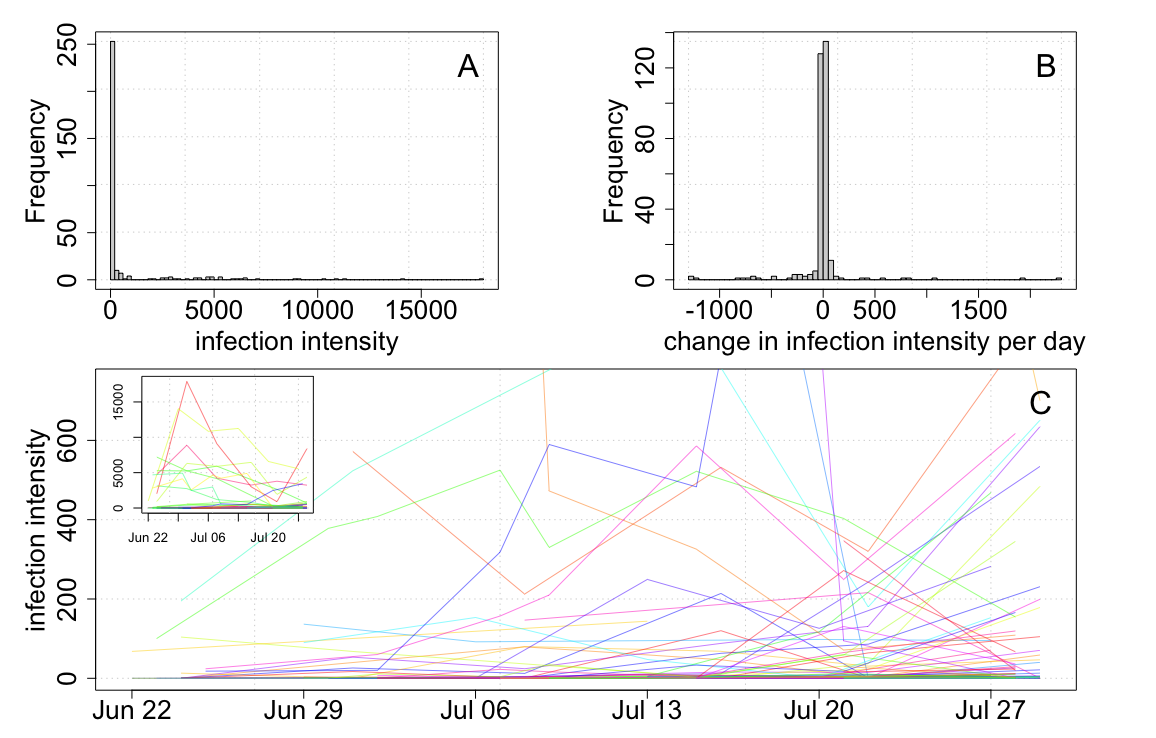
**

**Supplementary Figure 5: Infection intensity data**

Panel A shows the distribution of infection intensity measurements. Panel B shows the distribution of change in infection intensity per day, the response variable in the GAM fit to infer climate effects on infection intensity progression. Panel C shows observed trajectories of infection intensity, with each line representing an individual infected plant. The inset plot shows the same data with the y axis rescaled to show data for heavy infected plants. Colors are consistent across the main and inset plot.


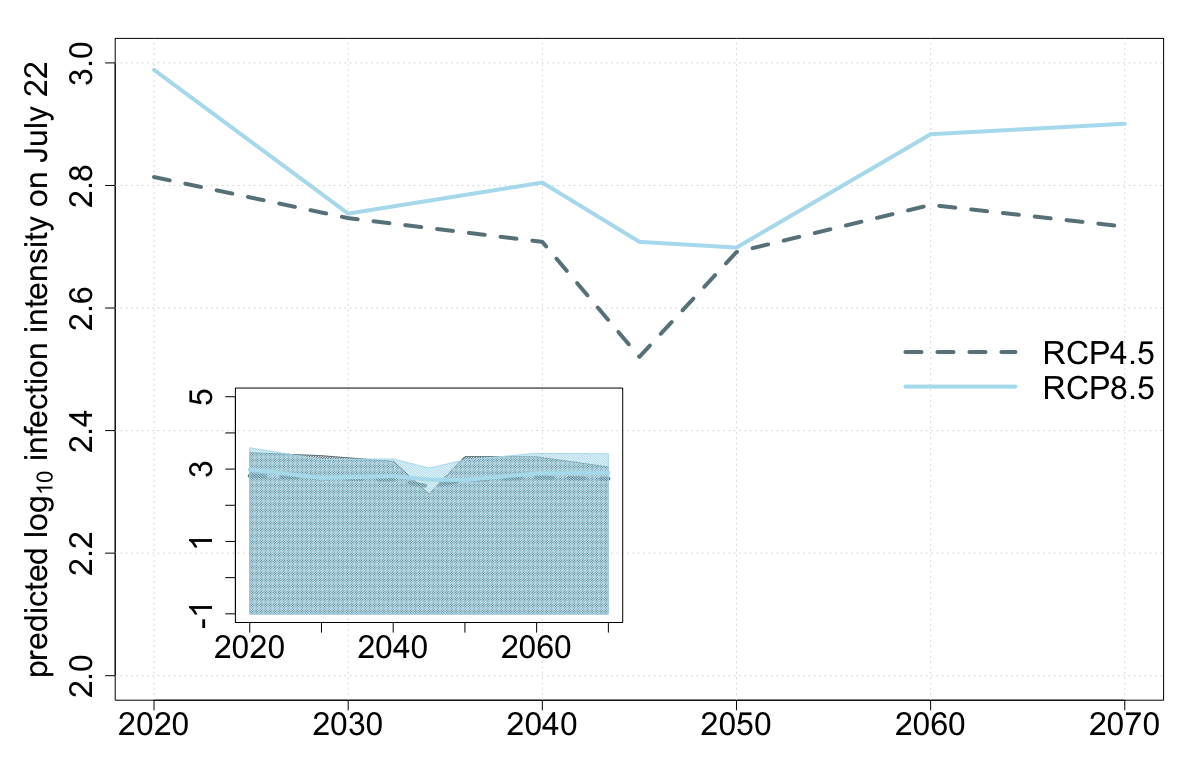


**Supplementary Figure 6: Patterns of end-of-simulation infection intensity across future climate scenarios**

We simulated 100 trajectories of infection intensity (for a 25cm tall plant starting with infection intensity 1) at the GM site for each of 12 future weather data sets encompassing the RCP 4.5 and 8.5 emissions scenarios and the years 2020, 2030, 2040, 2045, 2050, 2060, and 2070. Lines show the mean predicted infection intensity at the end of the simulation for each year and each emissions scenario. The inset plot shows the same results, with the range of the vertical axis expanded. Shaded regions span the 10% to 90% quantiles of predictions.

**
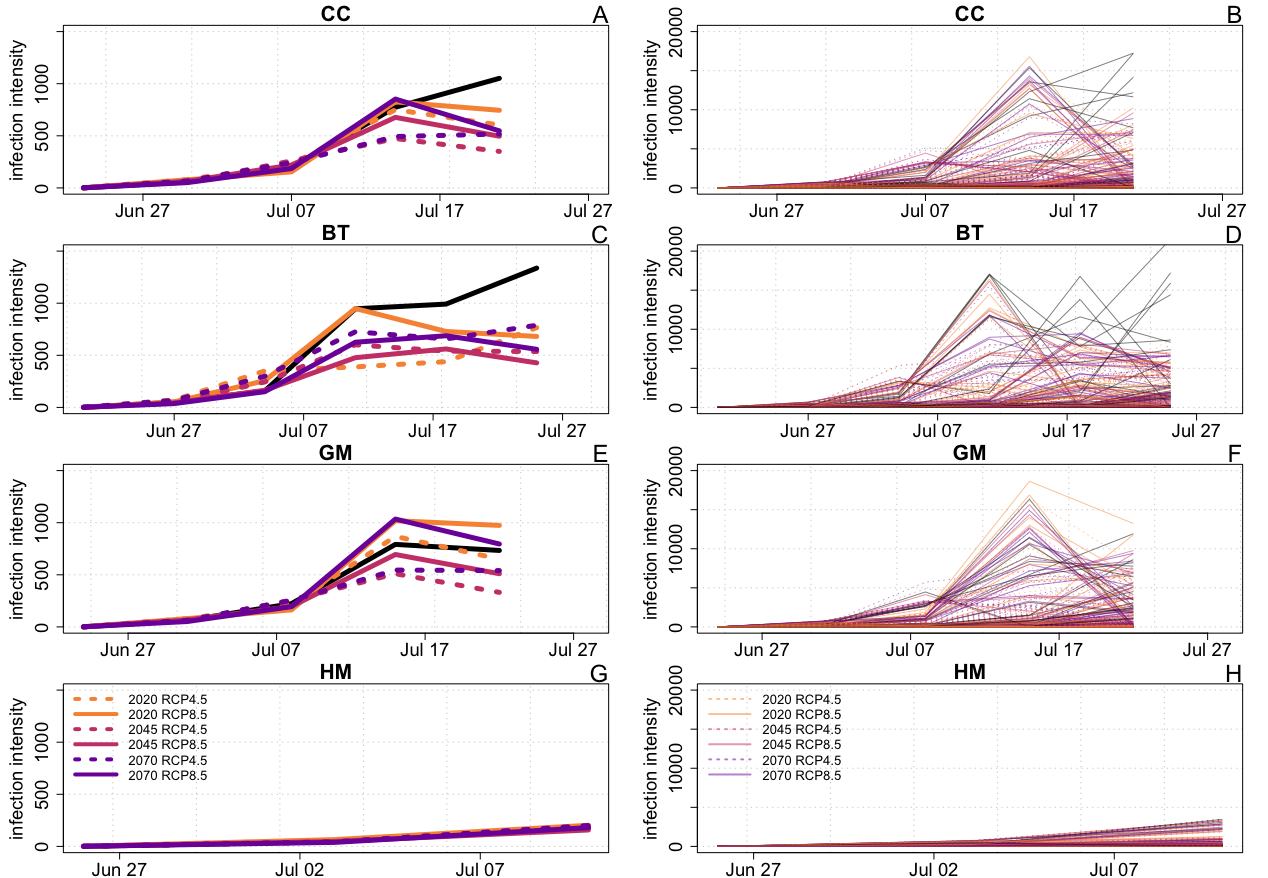
**

**Supplementary Figure 7: Simulated effects of future climate on infection intensity progression across study sites**

We simulated 100 trajectories of infection intensity progression (for a plant starting with a height of 25 cm and infection intensity of 1) at each site for six future weather data sets using the fitted GAM. Lines in panels A, C, E, and G show the mean of the trajectories simulated for each weather condition. Lines in panels B,D,F, and H show individual trajectories. Panels A and B correspond to the CC site, panels C and D correspond to the BT site, panels E and F correspond to the GM site (panel E is identical to Fig. 3J), and panels G and H correspond to the HM site.


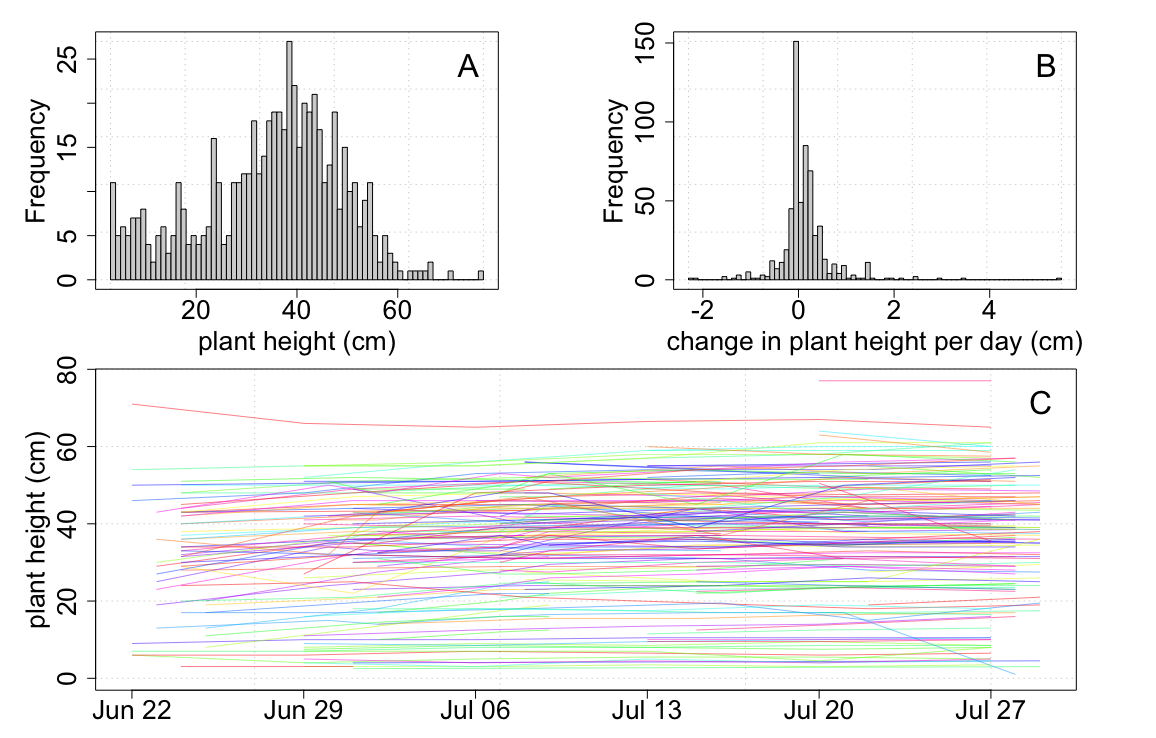


**Supplementary Figure 8: Plant growth data**

Panel A shows the distribution of pustule height measurements. Panel B shows the distribution of change in plant height per day, the response variable in the GAM fit to infer the effects of climate and infection on plant growth. Panel C shows observed height trajectories, with each line representing an individual plant.


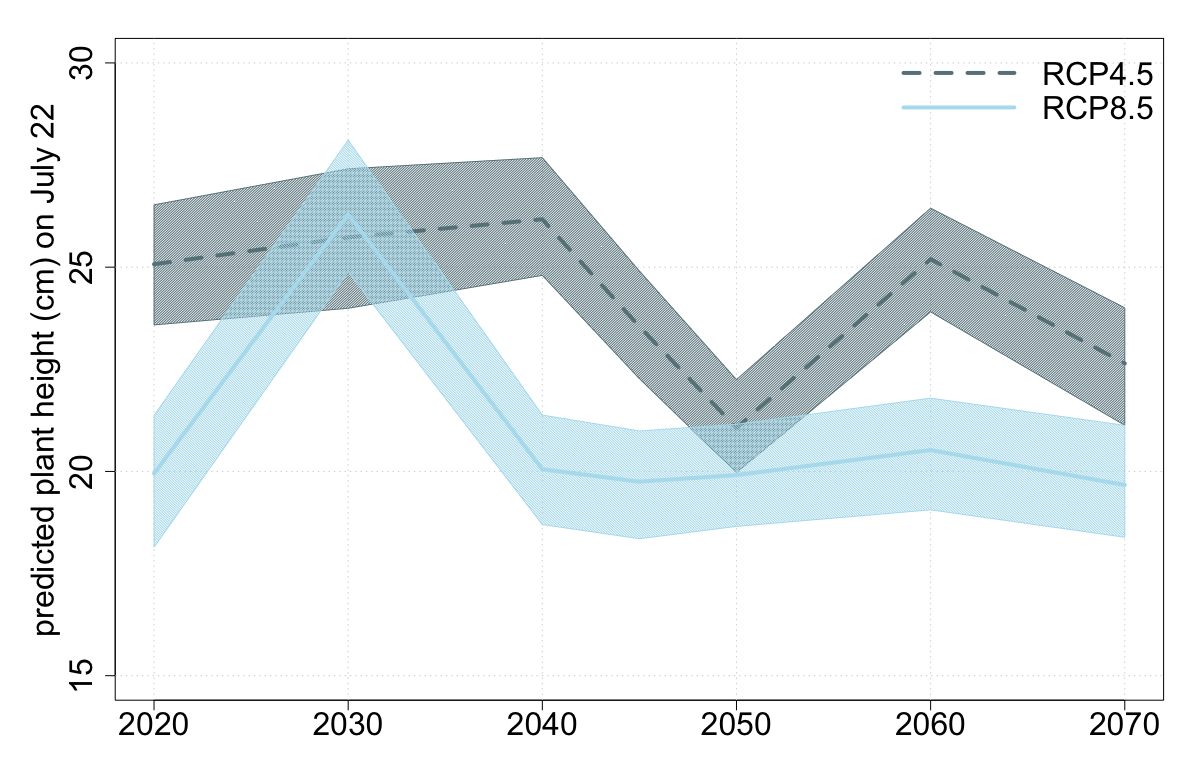


**Supplementary Figure 9: Patterns of end-of-simulation plant height across future climate scenarios**

We simulated 100 trajectories of plant growth (for a 25cm tall uninfected plant) at the GM site for each of 12 future weather data sets encompassing the RCP 4.5 and 8.5 emissions scenarios and the years 2020, 2030, 2040, 2045, 2050, 2060, and 2070. Lines show the mean predicted plant height at the end of the simulation for each year and each emissions scenario. Shaded regions span the 10% to 90% quantiles of predictions.


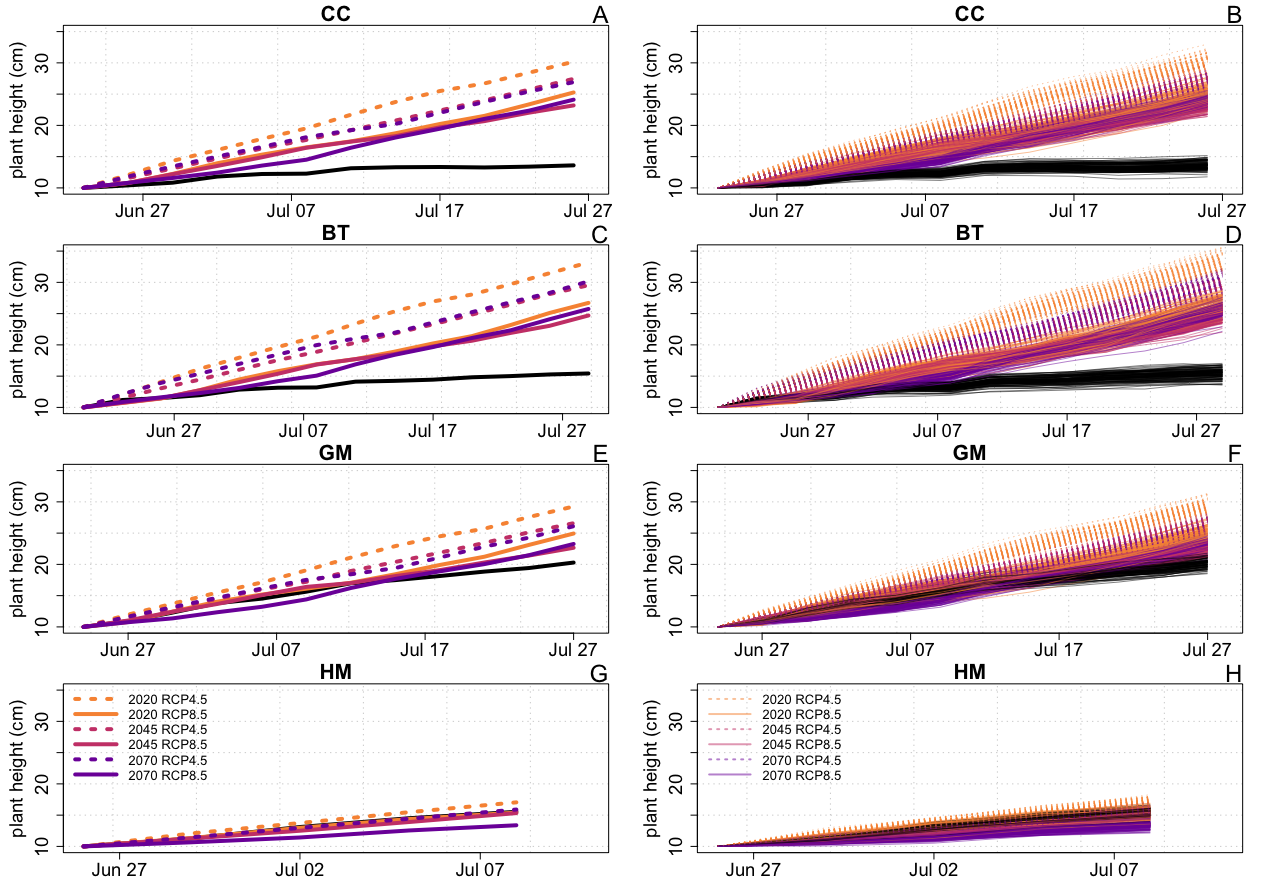


**Supplementary Figure 10: Simulated effects of future climate on plant growth**

We simulated 100 growth trajectories (for a plant starting with a height of 10 cm) at each site for six future weather data sets using the fitted GAM. Lines in panels A, C, E, and G show the mean of the trajectories simulated for each weather condition. Lines in panels B, D, F, and H show individual trajectories. Panels A and B correspond to the CC site, panels C and D correspond to the BT site, panels E and F correspond to the GM site (panel E is identical to Fig. 4J), and panels G and H correspond to the HM site.


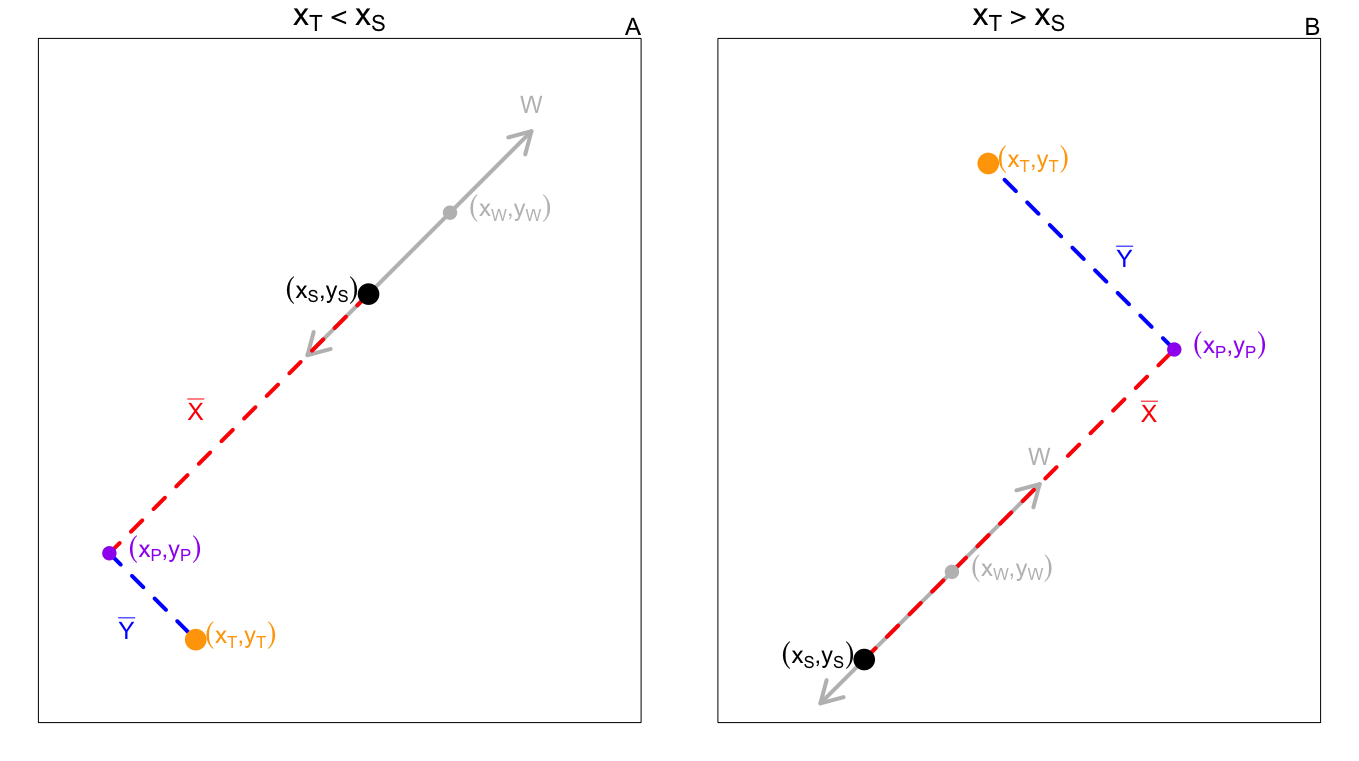


**Supplementary Figure 11: Calculating values of X and Y in the tilted gaussian plume model**

Figure S11 illustrates how the values of X and Y are found from the locations of the source and target plants and wind direction. Panel A shows an example where the target plant is to the left of the source plant, and panel B shows an example where the target plant is to the right of the source plant. Labels match the notation presented in the text.

**
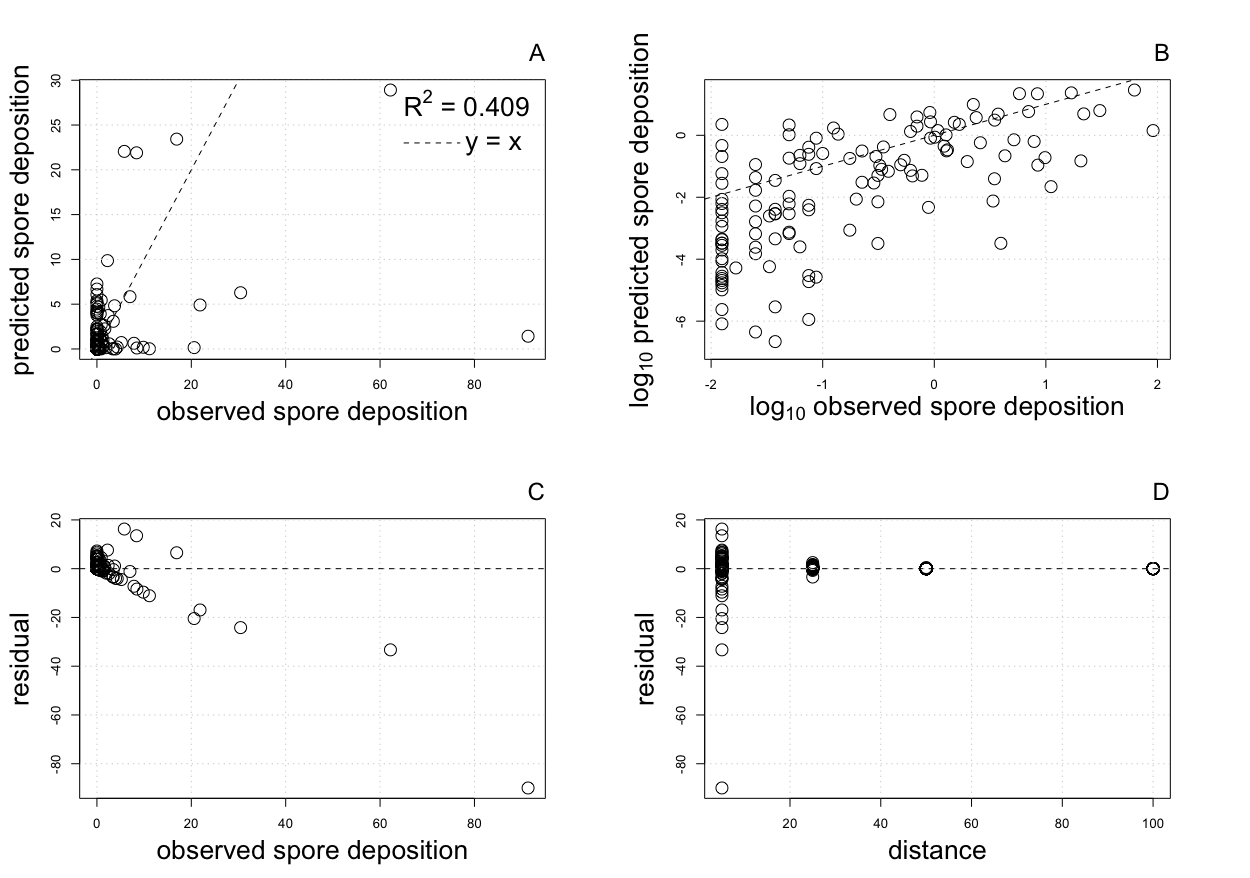
**

**Supplementary Figure 12: Tilted gaussian plume model diagnostics**

Panel A shows predictions generated by the TGPM plotted against spore deposition measurements from spore traps. The model explained 40.9% of the variance in the observed data. Panel B shows the same data as panel A with a log_10_ transformation applied to both observed and predicted values to illustrate how the model does a reasonable job of predicting the correct magnitude of spore deposition. In panel C, observed spore deposition is plotted against model residuals to illustrate that model performance was acceptable across the majority of the distribution of observed spore deposition values. Panel D shows the model residual plotted against the distance between a spore trap and the associated diseased focal plant. The mean of residuals is approximately zero for all distances, indicating that the model performs equally well at predicting spore deposition over small and large distances. The dashed lines in panels A and B show the y=x line. The dashed lines in C and D show y=0.

**
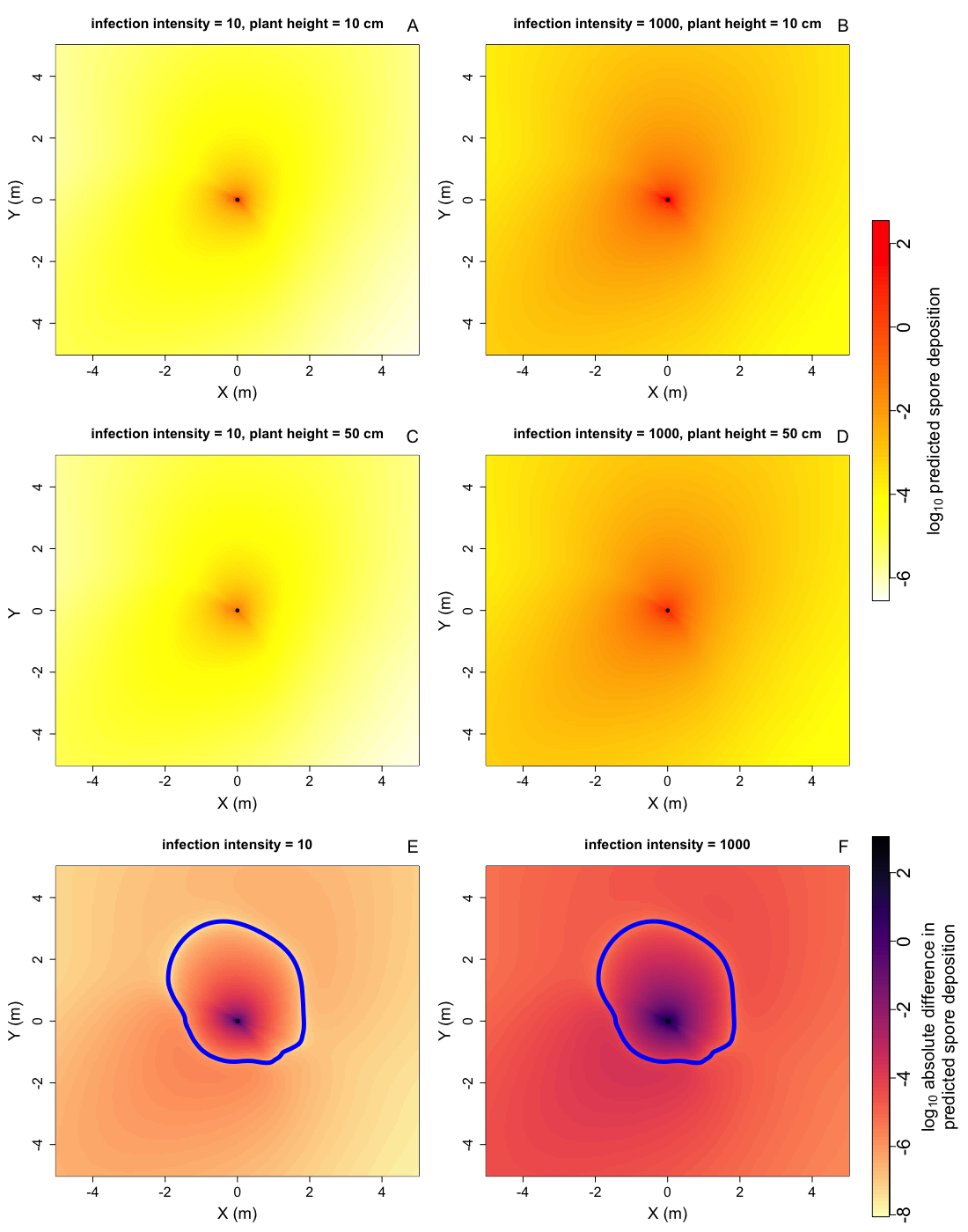
**

**Supplementary Figure 13: The effect of source plant height and infection intensity on spatial patterns of spore distribution predicted by the tilted gaussian plume model**

Figure S13 shows how the spatial pattern of spore distribution predicted by the TGPM depends upon the height and infection intensity of the source plant. In all panels, horizontal and vertical axes represent space. The source plant is located at {X=0,Y=0}. Predictions were generated using wind data collected at the GM site spanning noon on July 1st to noon on July 8th. In panels A and B, the plant’s height is assumed to be 10cm, and in C and D, the plant’s height is assumed to be 50cm. The plant is assumed to have infection intensity 10 in A and C, and infection intensity 1,000 in B and D. Colors in A-E show log_10_ predicted spore deposition. Panels E and F clarify the difference in patterns of spore distribution between panels A and C, and panels B and D. Colors show the base-10 logarithm of the absolute difference in predicted spore deposition for plants with height 10 cm and 50 cm. Infection intensity for both plants is assumed to be 10 in panel E and 1,000 in panel F. The blue line shows where the difference in total spore deposition is 0. The area enclosed by the blue line receives more spore deposition when plant height is 10 cm than when plant height is 50 cm. The area outside of the blue line receives more spore deposition when plant height is 50 cm than when plant height is 10 cm.


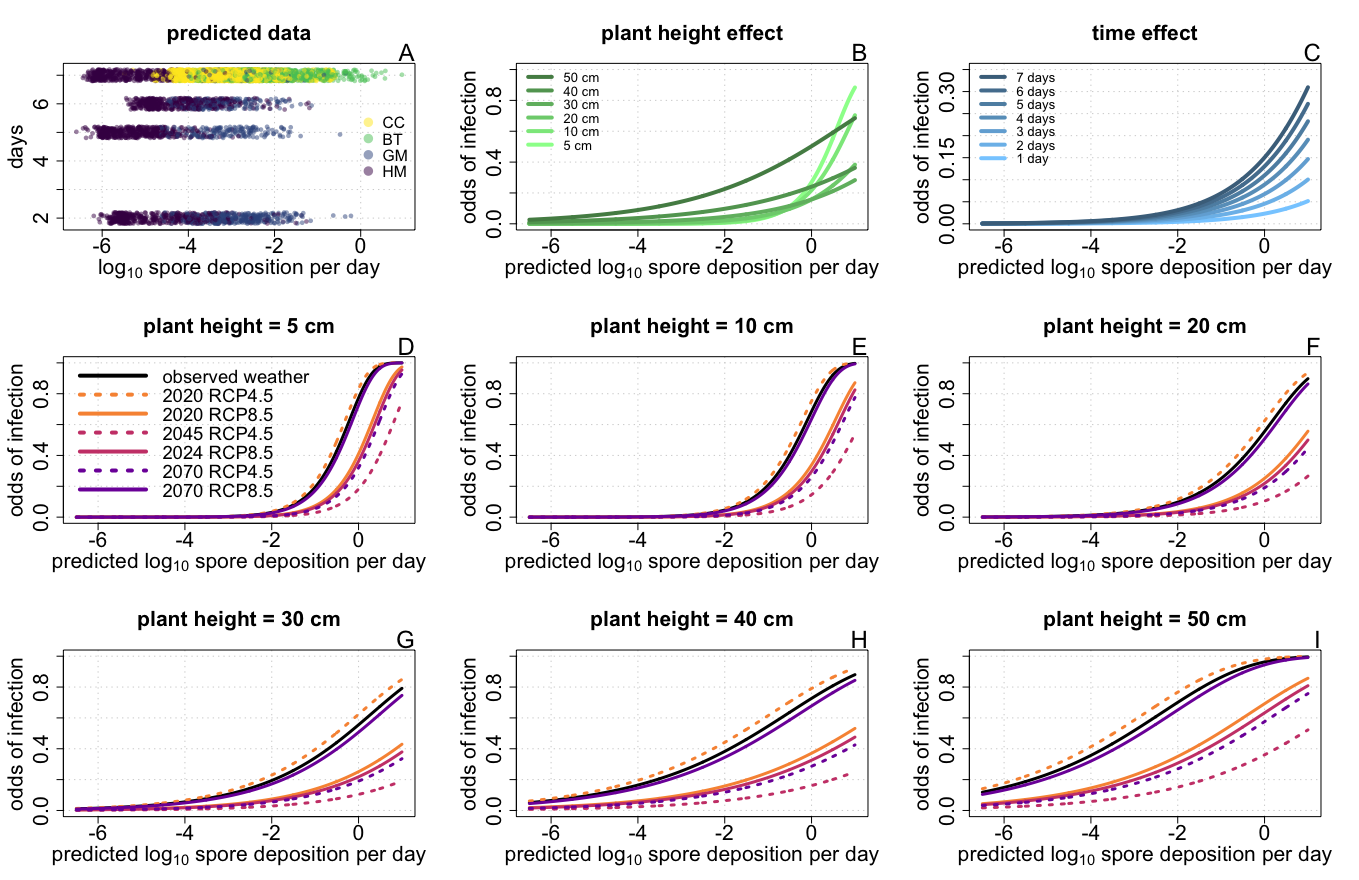


**Supplementary Figure 14: The effects of plant height and time on transmission odds**

Panel A shows an alternate visualization of the predicted spore deposition data presented in Fig. 5A, with the length of the period between observations of plant infection status plotted against log_10_ predicted spore deposition per day. Panels B and C show how the odds of infection inferred by the fitted GAM relate to plant height and observation time respectively. Predictions in panels B and C were generated using weather data observed at the GM site between July 7th and July 14th. Panels D-I show the relationship between log_10_ predicted spore deposition per day, plant height and the odds of infection inferred for different future climate scenarios. Plant heights are 5, 10, 20, 30, 40, and 50cm in panels D-I respectively. All predictions were generated for the GM site.


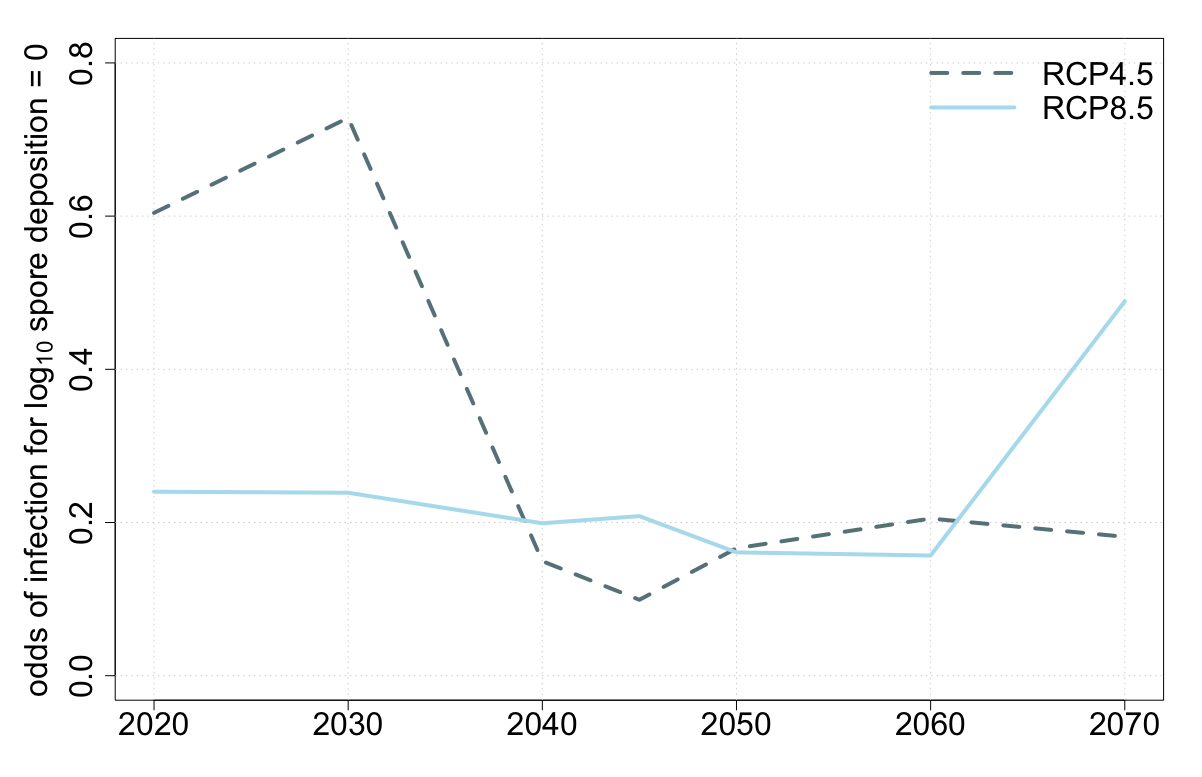


**Supplementary Figure 15: Patterns of predicted infection odds across future climate scenarios**

We simulated the odds of infection over the time period spanning July 7th-July14th for each of 12 future weather data sets encompassing the RCP 4.5 and 8.5 emissions scenarios and the years 2020, 2030, 2040, 2045, 2050, 2060, and 2070. In all cases, we considered a plant with height 25cm at the GM site and assumed log_10_ spore deposition per day = 0. Lines show the predicted odds of infection for each year and each emissions scenario.

**
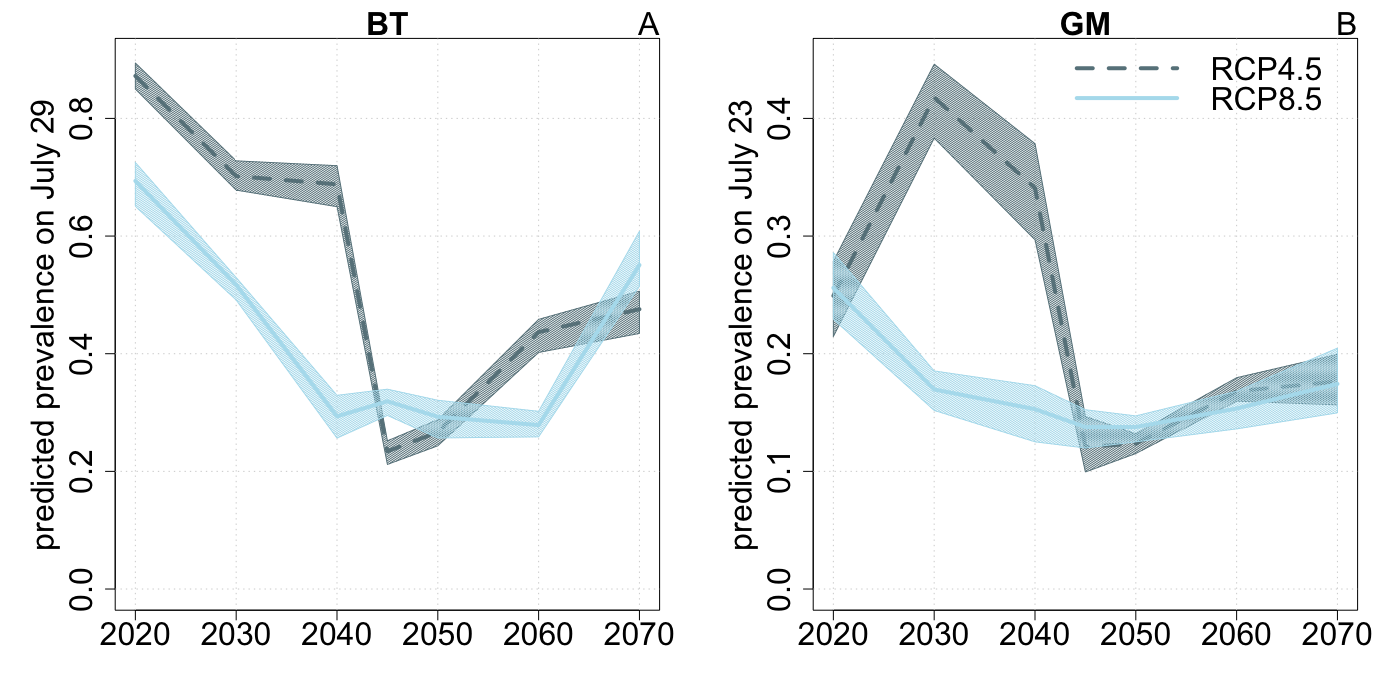
**

**Supplementary Figure 16: Patterns of end-of-simulation infection prevalence across future climate scenarios**

Using the spatiotemporal epidemiological model, we simulated 10 epidemics at the BT (panel A) and GM (panel B) sites for each of 12 future weather data sets encompassing the RCP 4.5 and 8.5 emissions scenarios and the years 2020, 2030, 2040, 2045, 2050, 2060, and 2070. Lines show the mean predicted prevalence at the end of the simulation for each year and each emissions scenario. Shaded regions span the 10% to 90% quantiles of predictions.

**Supplementary information references**

1. Bolton, D. The Computation of Equivalent Potential Temperature. *Mon. Weather Rev.* **108**, 1046–1053 (1980).

2. Morgan-Wall, T. rayshader: Create Maps and Visualize Data in 2D and 3D. (2022).
